# Biophysical Characterization of Viral and Lipid-Based Vectors for Vaccines and Therapeutics with Light Scattering and Calorimetric Techniques

**DOI:** 10.3390/vaccines10010049

**Published:** 2021-12-30

**Authors:** Natalia Markova, Stefan Cairns, Hanna Jankevics-Jones, Michael Kaszuba, Fanny Caputo, Jérémie Parot

**Affiliations:** 1 Malvern Panalytical Ltd., Enigma Business Park, Grovewood Road, Malvern, Worcestershire WR14 1XZ, UK; stefan.cairns@malvernpanalytical.com (S.C.); hanna.jankevics@malvern.com (H.J.-J.); michael.kaszuba@malvern.com (M.K.); 2 Department of Biotechnology and Nanomedicine, SINTEF Industry, 7465 Trondheim, Norway; fanny.caputo@lne.fr (F.C.); Jeremie.Parot@sintef.no (J.P.); 3 LNE—Centre for Scientific and Industrial Metrology, Avenue Roger Hennequin 29, 78197 Trappes, France

**Keywords:** vaccines, mRNA-LNPs, adeno associated viruses, quality control, particle size distribution, polydispersity, particle concentration, particle structure, rAAV stability, mRNA-LNP stability

## Abstract

Novel vaccine platforms for delivery of nucleic acids based on viral and non-viral vectors, such as recombinant adeno associated viruses (rAAV) and lipid-based nanoparticles (LNPs), hold great promise. However, they pose significant manufacturing and analytical challenges due to their intrinsic structural complexity. During product development and process control, their design, characterization, and quality control require the combination of fit-for-purpose complementary analytical tools. Moreover, an in-depth methodological expertise and holistic approach to data analysis are required for robust measurements and to enable an adequate interpretation of experimental findings. Here the combination of complementary label-free biophysical techniques, including dynamic light scattering (DLS), multiangle-DLS (MADLS), Electrophoretic Light Scattering (ELS), nanoparticle tracking analysis (NTA), multiple detection SEC and differential scanning calorimetry (DSC), have been successfully used for the characterization of physical and chemical attributes of rAAV and LNPs encapsulating mRNA. Methods’ performance, applicability, dynamic range of detection and method optimization are discussed for the measurements of multiple critical physical−chemical quality attributes, including particle size distribution, aggregation propensity, polydispersity, particle concentration, particle structural properties and nucleic acid payload.

## 1. Introduction

Appropriate analytical methods are needed to develop next generation vaccines and advanced medical products based on nanodelivery systems, such as recombinant adeno associated viruses (rAAV) and mRNA loaded lipid-based nanoparticles (mRNA-LNPs). Several key properties need to be efficiently and reliably measured and controlled to guide product design, inform optimization of the production process, and to determine stability and release specifications for the final product [1,2]. Among these key attributes are: (a) physical properties such as particle size, homogeneity and polydispersity, particle concentration, surface charge, phase transition temperatures, thermal stability; (b) chemical properties such as identity and quantity, impurities, degradation products, chemical stability of the nanoparticle components and of the delivered biomacromolecule (c) structural attributes such as the particle morphology, and the structural complex organization of the lipid and nucleic acids components and (d) biological efficacy and safety parameters such as bioactivity, immunogenicity, and potency [3,4,5,6,7].

The average diameter and size distribution are some of the most measured attributes during the development of delivery vectors for vaccines. These data are used for characterization and stability analysis starting from early development stages through process and formulation development all the way to process control, and batch release for clinical trials and marketed products [8,9].

Several analytical techniques can be employed to monitor the average size and the particle size distribution (PSD) of drug delivery vectors, such as dynamic light scattering (DLS), nanoparticle tracking analysis (NTA), multiangle dynamic light scattering (MADLS), and laser diffraction amongst others. Selection of the most suitable technology to monitor the sample will ultimately depend on the specific properties of drug delivery vectors, on their size and polydispersity, as well as what stage of the development process the measurements take place and what is the purpose of the measurement.

Information complementary to the light scattering based methods used in this study can be provided by imaging techniques (atomic force microscopy, AFM and transmission electron microscopy, TEM). Cryo-TEM is a golden standard technology to observe particle size, morphology and potentially drug loading. It enables particle visualization and helps to elucidate structure of complex molecular assemblies such as delivery vectors. However, accessibility of this imaging technique is often limited and the application to the samples as sensitive as LNPs poses additional challenges associated with the requirements for sample (particle) integrity during the analysis from the sample preparation to the measurement.

Small angle neutron scattering, or small angle X-ray scattering can also be complementary and can help to elucidate the structure of the lipid-based vectors and the distribution of the lipid components. However, as for Cryo-TEM, their accessibility, especially for the industry, can be limited.

Particle concentration is a less used attribute, mostly because the methodologies to measure particle concentration are still considered immature compared to particle sizing techniques. However, the information on the particle concentration can be very beneficial during design, formulation and processing of these delivery vectors, as it helps in monitoring yield and informing dosing. There are different methods to measure the particle concentration of soft organic particles that are reaching a more mature stage, including DLS, NTA and MADLS as well as separation techniques such as size exclusion chromatography (SEC) and asymmetrical field flow fractionation (AF4) coupled to static light scattering detectors or NTA [10,11,12]. Static light scattering detectors could be one of the following; multi-angle light scattering (MALS), right-angle light scattering (RALS) or low-angle light scattering (LALS), differing in the angle of detection and the analytics required to generate the parameters.

The properties of viral and LNP based vectors are determined by a very complex balance of the effects generated by the physical and chemical interactions between all their components [13,14,15]. The structural interactions between the active nucleic acids and the lipidic excipients generate a highly complex particle structure that dynamically changes, responding to modification of the physiological environment, e.g., responding to carrier protein interactions and to the changes of physiological pHs encountered in the biological tissues and in specific intracellular compartments. In the case of LNPs, it has been shown that faceted internal and surface structures form between the nucleic acids and the lipidic components of the carrier, increasing the efficiency of RNA transfection due to a superior capacity to trigger dynamic membrane fusion and endosomal escape [16].

If the determination of the particle structure per se is a very complex task, methods to measure the thermal stability and the evolution of surface charge of the nanoparticle vectors in biological environments could provide information about key structural properties and understand the relationship between these and the physical−chemical properties of the nanovaccines and their dynamic changes in biological environments. As practical examples, the thermal stability of the nanocarrier structure can be explored by measuring particle size with DLS thermal ramps and by differential scanning calorimetry (DSC). Moreover, changes in the surface charge of the nanocarrier responding to pH changes in physiological environments could be measured by performing a titration with electrophoretic light scattering (ELS).

Highly complex, multicomponent, multifunctional viral and non-viral delivery vectors such as mRNA-LNPs and rAAVs require fit-for purpose robust measurement methods for their characterization and the optimization of their stability [17,18], as summarized in Table 1. Complementary information on particle size, size distribution, concentration, charge, and chemical composition are required to reliably study the effects of pH, incubation time, temperature, and other stress conditions on the stability and function of these complex molecular assemblies.

This study reports detailed and comprehensive examples of the applicability and complementarity of DLS, NTA, MADLS, SEC-SLS, ELS and DSC for the biophysical characterization of viral and lipid-based vectors. The performances of these biophysical characterization techniques are compared based on model examples to inform researchers in the field and as part of an ongoing process to increase transparency and reproducibility of data and to ensure reliability of data interpretation. It is hoped that this work may help an informed discussion on the respective merits, drawbacks and synergies of the different measurement techniques.

The techniques used in this study are briefly described below. Further information can be found in the references provided in the text and in Table 1.

DLS is a non-invasive technique that measures the time-dependent fluctuations in the scattering intensity arising from a sample of particles or molecules undergoing Brownian motion. Auto-correlation analysis of these fluctuations allows calculation of the translational diffusion coefficients and subsequently the hydrodynamic size through the Stokes-Einstein relationship [30,31,32,33,34].

MADLS allows for a higher resolution size determination of multimodal samples, by using the three different detection angles (back, side and forward) and combining the information obtained into one angle-independent particle size distribution. Particle concentration measurements are an extension of MADLS and give the total particle concentration and the particle concentration for each mode present in a sample [35,36,37,38].

ELS is a technique for measuring the zeta potential of particle dispersions and macromolecular solutions. Zeta potential is the overall charge that a particle or macromolecule acquires in a particular medium and can be used to predict dispersion stability and provide information on the surface chemistry of the sample under investigation [39,40,41,42].

Size-exclusion chromatography (SEC) is a technique that separates molecules according to their hydrodynamic radius as they enter and exit the pores of a porous gel packing matrix in a column. A range of advanced detectors, such as static light scattering, UV, RI, and viscosity, allows for the measurement of absolute molecular weight, molecular size, intrinsic viscosity, branching, and other parameters [43].

Differential scanning calorimetry (DSC) detects changes in the apparent excess heat capacity resulting from rearrangements of structure and networks of inter- and intra-molecular interaction of a biomolecular sample in solution. DSC data inform on unfolding of proteins and protein domains [44], thermotropic phase transitions of lipids [45], thermal transitions of RNA and DNA molecules [46,47,48] and thermally induced disassembly of viruses—the complex associations of nucleic acids with proteins and, in some cases, lipids [49]. DSC is used in development and manufacturing of several commercial vaccines [50,51,52,53,54] and informs research and development of lipid-based delivery vectors, their equivalence and similarity [19,54,55]. In addition to multiple stability metrics, DSC provides a fingerprint of Higher Order Structure defined by a range of intra- and inter-molecular interactions in a sample.

## 2. Materials and Methods

### 2.1. Materials

Commercial rAAV samples (rAAV5, rAAV2 and rAAV9) were purchased as nominally empty and nominally full rAAVs from Virovek (Hayward, CA, USA). These rAAV capsids are referred to as empty and full throughout the rest of the paper. From information provided by the supplier, the rAAV vectors were purified through two rounds of CsCl-gradient ultracentrifugation followed by sterile filtration of the nominally empty and nominally full fractions.

The rAAV samples were used as received following the manufacturer’s specifications for purity and titer. The full rAAVs contained pFB-GFP ssDNA which consisted of 2544 nucleotides with a known molecular weight (Mw) of 785,000 g/mol. The sample had a defined viral titer of 2.5 × 10^13^ viral genome per mL (vg/mL) as calculated by qPCR. The disperse phase of the samples was PBS containing 0.001% Pluronic F-68 (Virovek, Hayward, CA, USA).

The sample of enveloped Modified Vaccinia Ankara virus was generously provided by Leukocare AG (Martinsried, Germany) and was used as received. The sample had a viral titer of 2.19 × 10^9^ PFU/mL. The disperse phase of the sample was 50 mM TRIS buffer pH 9.

mRNA-LNPs were provided by SINTEF Industri (Department of Biotechnology and Nanomedicine, Trondheim, Norway), and made as described elsewhere [12]. In short, two formulations of mRNA-LNPs were synthetized using Nanoassembler (Precision Nanosystems) with stock lipid solutions of cholesterol, DSPC, and PEG2000-DMG (from Avanti Polar) at 10 mg/mL and of MC3 and SM102 (from Organix) at 20 mg/mL, respectively called LNP1 and LNP2. FLuc CleanCap^®^ FLuc mRNA (5moU) used to formulate the LNPs in this study was purchased from Tebu-Bio (Roskilde, Denmark).

The total lipid content and the content of the encapsulated mRNA in the final mRNA-LNP1 and mRNA-LNP2 preparations was determined as described in [12].

The liposomes used were Formumax HSPC/CHOL liposomes (Cat No.: F0104, Batch No.: 08081701) and were diluted with Gibco PBS at pH 7.2 (Cat No.: 20012019, Lot No.: 1880350) using gravimetric dilution protocol. All samples to be used for concentration measurements were prepared gravimetrically using a 5 decimal balance.

### 2.2. Methods

#### 2.2.1. Dynamic Light Scattering (DLS)

Dynamic light scattering measurements were made with a Zetasizer Ultra (Malvern Panalytical Ltd., Malvern, UK) using a He-Ne laser at a wavelength of 633 nm and maximum power of 10 mW. Five repeat measurements of each sample were made, using backscatter detection and a low-volume quartz batch cuvette (ZEN2112 Malvern Panalytical Ltd., Malvern. UK). The instrument settings were optimized automatically by means of the ZS XPLORER software (Malvern Panalytical Ltd., Malvern, UK).

#### 2.2.2. Multi-Angle Dynamic Light Scattering (MADLS)

Multiangle dynamic light scattering (MADLS) particle concentration measurements were carried out using a Zetasizer Ultra (Malvern Panalytical Ltd., Malvern, UK) using a He-Ne laser at a wavelength of 633 nm and maximum power of 10 mW. All experiments were carried out at 25 °C unless stated otherwise. The instrument has a tolerance of 0.1 °C. All measurements were carried out in a low-volume quartz batch cuvette (ZEN2112 Malvern Panalytical Ltd., Malvern. UK). The instrument settings were optimized automatically by means of the ZS XPLORER software (Malvern Panalytical Ltd., Malvern, UK).

The particle optical properties used for the measurements were calculated assuming solid sphere and using the data from nucleoside analysis for determination of RNA content and lipid content, respectively [10]. The values used in this study for mRNA-LNP1 and mRNA-LNP2 batch 1 were particle refractive indices of 1.47, and for LNP2 batch 2 and batch 4, 1.46, respectively. An absorption of 0.001 was used for all particles. The scattering intensity of the dispersants used for each of the different vectors, was measured in back scatter detection (non-invasive backscatter NIBS) and were in the region of 80 to 90 kilo counts per second (kcps), respectively, and used for the particle concentration measurements. The dispersant viscosity was assumed to be that of water, as the lipid nanoparticles were dispersed in PBS.

#### 2.2.3. DLS Thermal Ramps

Thermal ramps, covering a temperature range of 10 to 75 °C with 1 °C increment, were carried out using a Zetasizer Ultra (Malvern Panalytical Ltd., Malvern, UK) using a He-Ne laser at a wavelength of 633 nm and maximum power of 10 mW. Size measurements were collected at every 1 °C increment using backscatter detection and a particle concentration measurement taken every 5 °C using a low-volume quartz batch cuvette (ZEN2112 Malvern Panalytical Ltd., Malvern. UK).

#### 2.2.4. Nanoparticle Tracking Analysis (NTA)

Nanoparticle tracking analysis measurements (Table 2) were carried out using a Nanosight NS300 instrument (Malvern Panalytical Ltd., Malvern, UK) (405 nm). All measurements were performed with temperature control at 25 °C. The camera setting, and the focus were set automatically by the software; and 5 repeat measurements of 60 s were performed for each sample. The sample was loaded into a dry flow cell and at least 500 µL were flushed in the flow cell before measurements. The syringe pump was set so that the particles traversed the measurement region in 10 to 12 s.

All buffers used were checked for particles at cameral level 16. As no particles were found, no further filtration was used.

#### 2.2.5. Electrophoretic Light Scattering (ELS)

Electrophoretic light scattering (ELS) measurements of the lipid nanoparticles samples were measured on a Zetasizer Ultra Red instrument (Malvern Panalytical Ltd., Malvern, UK) equipped with a He-Ne laser at a wavelength of 633 nm and maximum power of 10 mW. Amounts of 700 µL of the samples were introduced into a folded capillary cell (DTS1070 Malvern Panalytical Ltd., Malvern, UK). The duration of the zeta potential measurements was manually set up with 10 sub runs used per measurement. All other measurement settings were optimized automatically by the ZS XPLORER software. All measurements were made at 25 °C and consisted of 3 zeta potential measurements followed by 5 DLS size measurements using backscatter detection. A 60 s delay between each zeta potential measurement was used to minimize Joule heating and polarization effects. The field strength used was approximately 8 V/cm and the measured electrophoretic mobilities were converted into zeta potentials using the Smoluchowski approximation [39,40,41].

pH titrations of the mRNA-LNPs prepared in 10 mM NaCl (10 mL sample volume) were performed with the multipurpose titrator 3 (MPT3) accessory, degasser and Zetasizer Ultra Red at a temperature of 25 °C. Each pH titration contained three segments with different pH step sizes. The 1st segment covered pH 9.5 to 7.5 with pH 1 steps, the 2nd segment was from pH 7.5 to 4.5 with pH 0.5 steps and the 3rd segment pH 4.5 to 2.5 with pH 1 steps. The titrants used for changing the sample pH were 0.25 M and 0.025 M HCl and 0.25 M NaOH, respectively. A stirrer bar was active during the whole measurement to improve pH stability. Five zeta potential measurements were taken at each pH point to check for result repeatability. Zeta potential measurement duration was manually defined and consisted of 20 sub runs per measurement. All other measurement settings were optimized automatically by the ZS XPLORER software.

#### 2.2.6. Size-Exclusion Chromatography Static Light Scattering (SEC-SLS)

Size-Exclusion Chromatography Static Light Scattering (SEC-SLS) Chromatographic separation of the (i) rAAV and (ii) mRNA-LNP samples was achieved using the following chromatographic conditions: (1) SEC column Sepax SRT 500 4.6 × 300 mm with an iso-cratic flow rate of 0.5 mL/min and phosphate-buffered saline or 0.01 M NaH2PO4 + 0.01 M Na2HPO4 + KCl 0.35 M pH 6.6 as the mobile phase; (2) an OMNISEC system (Malvern Panalytical Ltd., Malvern, UK) consisting of an OMNISEC RESOLVE (pump, autosampler, and column oven) and an OMNISEC REVEAL (refractive index, UV/Vis- photodiode array detection (PDA) and static light scattering (SLS) detector, comprised of two observation angles right angle (90°) and low angle (7°)) was used to acquire the sample chromatograms. The samples were maintained at 4 °C in the autosampler prior to injection. The column oven and detector module were maintained at a constant 30 °C during this work.

Compositional analysis method [56] was employed to determine the concentration and molecular weight of two distinct components within a sample. For the compositional analysis to work, it is necessary to know the refractive index increment (dn/dc) and extinction coefficient (dA/dc) of both components. In general, the vaccine is divided into the delivery vehicle and the genomic contents or API. For the example of rAAVs discussed below, the dn/dc values of the capsid and the ssDNA are well known. The dA/dc for the capsid is measured using OMNISEC, and the dA/dc for the ssDNA is calculated from the sequence. The values used for these components were: dn/dc_capsid rAAV5_ 0.185, dn/dc_capsid rAAV9_ 0.185, dn/dc_capsid rAAV2_ 0.185, dn/dc_DNA_ 0.170, dn/dc_LNP_ 0.13, dn/dc_Luc-RNA_ 0.170, dA/dc_capsid rAAV5_ 1.33, dA/dc_capsid rAAV9_ 1.22, dA/dc_capsid rAAV2_ 1.33, dA/dc_DNA_ 30, dA/dc_Luc-RNA_ 35.

The compositional analysis in the OMNISEC V11.30+ software determines the concentration of each component present at every point in the chromatogram. The two equations for RI and UV observables below contain the two variables conc_capsid_ and conc_DNA_ and may be solved for one of these two unknowns followed by the other as one would solve for any simultaneous equation. They are simultaneous as the concentrations of each component in both equations are equivalent when the delay volume between the detectors is accounted for (note this is done by the software).

RI = conc_capsid_ × dn/dc_capsid_ + conc_DNA_ × dn/dc_DNA_, (1)

UV = conc_capsid_ × dA/dc_capsid_ + conc_DNA_ × dA/dc_DNA_, (2)

Total particle (Equation (3)), full particle (Equation (4)), and empty particle (Equation (5)) concentration can all be obtained using the following equations:

C_total_ = (conc_capsid_ × N_A_)/(Mw_capsid_), (3)

C_full_ = (conc_DNA_ × N_A_)/(Mw_seq DNA_), (4)

C_empty_ = C_total_ − C_full_, (5)

%full rAAV = 100 × C_full_/C_total_, (6)

cp/vg ratio = C_total_/C_full_, (7)

where conc_capsid_ is the concentration of the capsid in mg/mL as calculated, N_A_ is Avogadro’s number, Mw_capsid_ is the molecular weight (g/mol) of the capsid as calculated, conc_DNA_ is the concentration of the DNA in mg/mL as calculated, and Mw_seq DNA_ is the molecular weight of the ssDNA from the sequence. Therefore, using these calculated particle concentrations, the percentage of full rAAV was derived for rAAVs of three different serotypes: rAAV2, rAAV5 and rAAV9. The compositional analysis of SEC–SLS data in this study is based on a simplified model where capsids are treated as empty or full species only.

#### 2.2.7. Differential Scanning Calorimetry (DSC)

MicroCal PEAQ DSC automated (Malvern Panalytical, Northampton, MA, USA) was used for analysis of thermal stability of empty and full rAAV5 samples and preparations of mRNA- LNP1, mRNA-LNP2 and mRNA solution. For the analysis, 325 μL aliquots of the samples and matching buffer solutions were loaded onto a 96-well plate, covered with a silicon seal, and placed into the PEAQ DSC plate stacker thermostated at 10 °C.

The thermal scans were performed in the range from 4 °C to 100 °C or from 4 °C to 130 °C at a scan rate of 60 °C/h. In between the sample measurements, the sample and the reference cells of the PEAQ DSC instrument were automatically cleaned with 14 *v*/*v*% Decon 90 solutions following a pre-set SCAN cleaning procedure replicating the scanning conditions used in the measurements and including a thorough rinse with Milli-Q^®^ filtered water.

The data were analyzed with dedicated PEAQ DSC Analysis software (Malvern Panalytical, Northampton, MA). For analysis, sample thermograms were normalized for the sample concentration and corrected for the instrument baseline by subtraction of the corresponding buffer-buffer scan. Finally, the sample thermograms were automatically baseline-corrected following extrapolation of pre- and post-transition baselines with a spline function. The resulting normalized and baseline-corrected DSC traces of rAAV5, mRNA-LNPs and free mRNA samples were analyzed for T_onset_, T_m_, total heat effect and enthalpy of transition, Δ*H*_tr_.

## 3. Results

**Figure 1:**
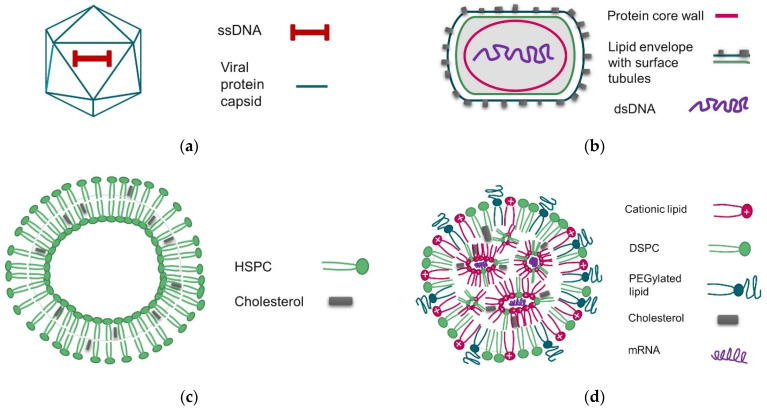
Schematic representation of the basic structures and compositions of nanoparticles characterized in this study. (**a**) rAAV, (**b**) MVA virus, (**c**) HSPC/CHOL liposome, (**d**) mRNA-LNP.

To explore the capabilities of calorimetric and light scattering techniques for characterization of key attributes of viral and non-viral delivery vectors, a diverse range of mRNA-LNP, rAAV5 and liposomal samples covering a broad size and particle concentration range and carrying DNA and RNA payload were analyzed (Figure 1 and Table 3).

### 3.1. Size Distribution and Sample Polydispersity

#### 3.1.1. What Sizing Techniques Work for Vectors >50 nm?

**Figure 2:**
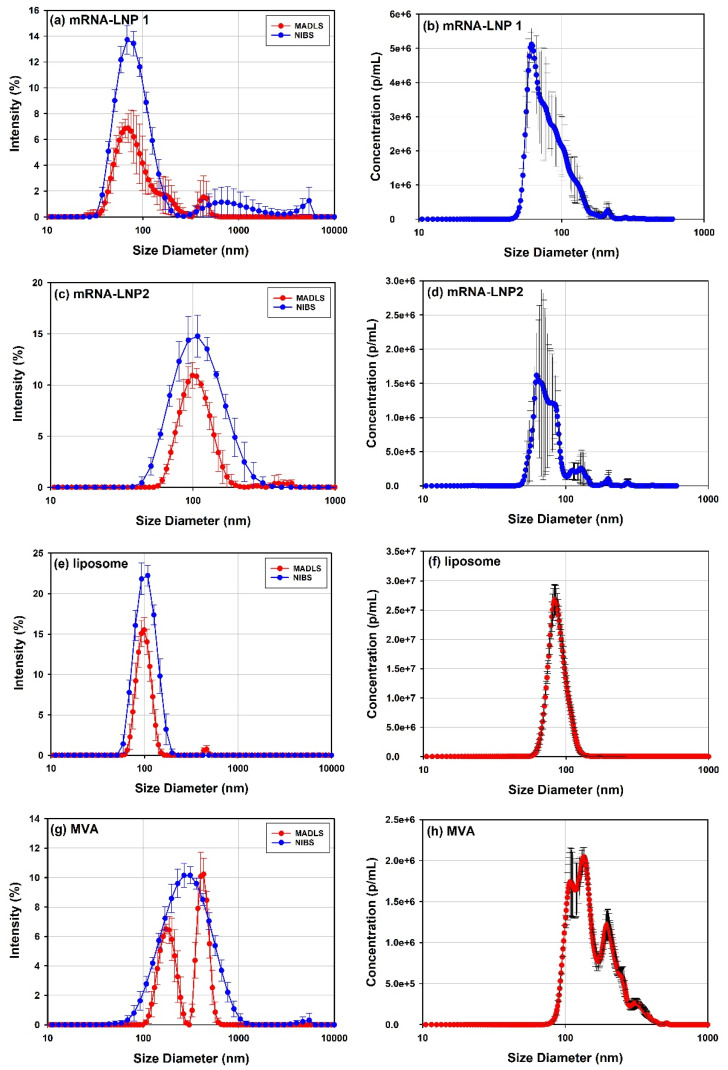
Example of size distributions measured with DLS measured through non-invasive back scattering (NIBS) detection and MADLS (**a**,**c**,**e**,**g**) and NTA (**b**,**d**,**f**,**h**) are shown for a variety of delivery vectors such as mRNA-LNP 1 (**a**,**b**), mRNA-LNP2 (**c**,**d**), liposome (**e**,**f**) and MVA (**g**,**h**) vectors.

In Figure 2, the PSD profiles of several viral and LNP-based delivery vectors measured by DLS and MADLS (Figure 2a,c,e,g) and NTA (Figure 2b,d,f,h), are compared to demonstrate the applicability and limitations of these approaches [30,57,58].

The use of these three analytical techniques help cover a wider size range with different resolution. DLS has the lowest resolution but provides a good indication of what particle sizes are present in the sample, as seen from its overlap with the other techniques (Figure 2).

MADLS has improves measurement resolution compared to DLS [38] but not as high as NTA, as proven by the measurements of mRNA-LNP1 (Figure 2a) and MVA (Figure 2g) where it can identify additional populations present in the sample compared to DLS. However, MADLS PSD for the MVA sample does not show the full lower size range as seen with NTA. The difference in detection capability shown by MADLS for mRNA-LNP1 and MVA, where the former is fully captured but the latter is lacking detail of the lower end of the PSD, is due to the relative amount of light from the different populations present in the sample. For mRNA-LNP1, the aggregate peaks contribute 21% of the scattered light intensity, whereas for MVA, the largest aggregate peak contributes 51% of the scattered light, as determined from the peak areas for these populations. The aggregate peak in MVA is also much larger in size than the aggregate population in mRNA-LNP1. The ability of MADLS to detect populations of smaller sizes in the presence of larger aggregates is critically dependent on (i) the relative fraction of larger aggregates in the sample and (ii) their size in comparison to the size of the species in the main population of smaller particles. It is therefore not possible to specify a unique value for a maximum permissible fraction of aggregates which would allow for robust detection of smaller sized populations in a polydisperse sample by MADLS.

In the case of mRNA-LNP1, mRNA-LNP2 and MVA, NTA (Figure 2b,d,f,h) has improved resolution power compared to MADLS. This demonstrates the value of using NTA over DLS for the analysis of polydisperse samples. NTA most often requires significant dilutions of the sample, and it is important to verify sample stability at these conditions, which is typically done by replicate measurements for an extended time period. mRNA-LNP2 indicated sample instability (data not shown) by a reduction in sample concentration across replicate measurements. This is one of the reasons for the increased variability across the PSD compared to mRNA-LNP1.

The population size parameters for the different samples, shown in Figure 2, are recorded in Table 4, where the z-average is the intensity-weighted mean size of the full sample [30] (including any aggregates). This intensity weighting makes it a sensitive parameter in highlighting the presence of aggregates in the sample. This can be illustrated by comparing the mRNA-LNP2 and the liposome samples, which both have very low amounts of aggregates as identified from the peak areas of the main population (98% for both). The majority of the light is therefore scattered by the main population, and the z-averages and the peak mean sizes from MADLS for these samples are very similar and within measurement variation.

In the case of mRNA-LNP1 and MVA samples, less light is scattered by the main population, 79% and 49%, respectively, based on peak areas, where the z-average sizes are between the peak sizes as determined by MADLS due to intensity weighting.

#### 3.1.2. What Sizing Techniques Work for Vectors <50 nm?

**Figure 3:**
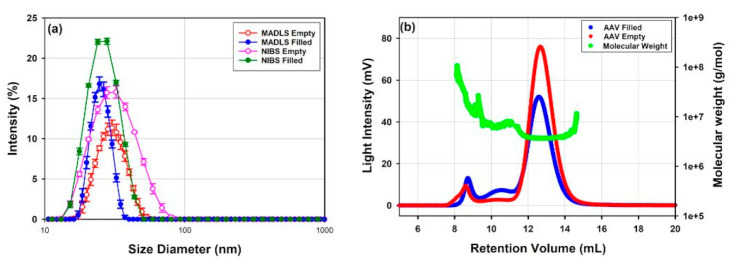
(**a**) Non-invasive backscatter DLS and MADLS intensity PSDs of rAAV5 samples batch 1 that are either full or empty. (**b**) shows how SEC separation of the same samples ahead of static light scattering detectors increases size distribution resolution compared to MADLS.

AAV vectors, which are relatively small delivery vectors of around 20 to 30 nm in diameter, are well characterized by DLS and MADLS (Figure 3a). Two samples are shown of the same rAAV5 serotype, one without payload (empty) and one with transgene payload (full). NTA cannot detect the main population, as it is below its lower size detection limit, and only shows the presence of small aggregates in the sample (data not shown).

For an in-depth characterization of the different size populations of polydisperse delivery vectors, a separation method such as SEC or FFF prior to the size measurement can be used to improve the resolution of identified populations and then measured with inline detectors such as RI/UV and SLS. In Figure 3, DLS, MADLS and SEC-SLS measurements of rAAV5 capsids filled with ssDNA or empty capsids are compared. Both DLS and MADLS particle size distribution profiles of the full rAAV5 sample seem slightly shifted to smaller sizes compared to the empty rAAV5 particle size distribution (Table 4). DLS and MADLS both measure hydrodynamic size, and therefore any additional content such as a transgene inside the viral capsid is unlikely to be detected as a size change. However, the shift of the particle size distribution of the empty rAAV5 may indicate the presence of small aggregates that are not completely resolved from the main capsid population. The use of SEC-SLS confirms that the empty rAAV5 sample contains a larger percentage of aggregates if compared to the full rAAV5 sample, see Figure 3b and Table 5. The molecular weight information for the dimer and aggregates of the rAAV5 full sample, despite following the same elution times as those observed for the rAAV5 empty sample (Figure 3b), were found to be highly variable as the concentration approached the lower limit of quantification.

The lower limit of quantification for SEC-SLS is directly influenced by the separation conditions and the resulting dilution of the injected sample mass. To circumvent this, the chromatography conditions were developed. The Superose 6 was changed for a Sepax SRT 500 4.6 × 300 mm to minimize sample dilution as it passes through the SEC column. From Table 5, the molecular weights calculated for the dimer of both the full and empty sample show the expected values and are less variable despite analyzing a 2-fold lower concentration.

This example shows how MADLS could be useful as a pre-screening indicator of the presence of small amounts of aggregates. However, it cannot resolve close populations such as monomers and small oligomers. If further information about the amount of these aggregates present is needed, SEC-SLS may be used to quantify the fraction of the main population to track batch-to-batch variability and the aggregation propensity of different formulations. As shown above, batch-to-batch comparisons may be made between the samples. The use of SEC-SLS allowed for the comparison of what oligomeric states are present in the samples, as each population in the sample may be distinctly identified and quantified. From Table 5, the comparison between the two batches shows a reduced fraction of aggregates in both the empty and full samples and increased percentage monomer for batch 2 compared to batch 1.

The particle size distribution data for mRNA-LNP1 and 2, liposomal sample and viral samples rAAV5 and MVA demonstrate how the selection of an appropriate technology to measure particle size distribution ultimately depends on the particle sizes and the distribution shape, and how orthogonal measurements are important to fully assess the particle size distribution during development enabling the identification of the most appropriate size measurements for the development stage.

#### 3.1.3. Viral and Non-Viral Vector Polydispersity by DLS, MADLS, NTA and SEC-SLS

The heterogeneity or polydispersity of drug delivery vectors and vaccines may be an important parameter to assess sample stability in the drug development process. As an example, Table 6 reports the polydispersity indicators measured by each analytical technique for each of the samples shown in Figure 2 and Figure 3. The parameter used to assess polydispersity varies depending on which technique is applied [59]. Depending on the measurement needs, the polydispersity of the full sample including all populations may be reported, or the polydispersity of an identified population among many may be used.

#### 3.1.4. What Can Be Said about Sample Polydispersity?

The measure of polydispersity associated with the whole particle size distribution is used to describe the presence of aggregates or agglomerates. Useful parameters are the polydispersity index (PdI) from the DLS cumulants analysis [30] and Span [60] calculated either from volume transformation of DLS non-negative least squares (NNLS) data or MADLS data, or NTA size distributions (Table 4). The polydispersity index can also be used to calculate a %Polydispersity (%Pd), (equal to the √PdI × 100). A sample with polydispersity index below 0.04, which translates into 20%Pd, is considered a monodisperse sample. The only samples reported in Table 4, that qualify as monodisperse, according to these rules are the full rAAV5 and liposome samples.

The other measure for sample polydispersity, often used in laser diffraction measurements [], is span ((D90 − D10)/D50) giving an indication of the broadness of the distribution. The closer the value is to 0, the more monodisperse the population is. For an indication of the polydispersity via Span for DLS and MADLS results, a transformation to a volume distribution is required, whereas for NTA, the calculation is based on the number PSD.

The full rAAV and liposomes samples that were defined as monodisperse by the PdI data also show span values well below 1 (around 0.4 for the MADLS and NTA data, Table 4). The polydisperse samples, with clear presence of second populations, such as MVA and LNP1 have a span around 1 for both their MADLS and their NTA size distributions. Unsurprisingly, the spans calculated from the DLS size distributions are the largest, as these are inherently broader distributions.

In SEC-SLS, the sample polydispersity is often referred to as % monomer or % fraction of sample [61], in terms of the amount of sample in the main population compared to the total amount of sample from all population peaks, including larger aggregates and smaller fragments. The two rAAV5 samples, as shown in Table 5, demonstrate different fractions of sample, with the empty sample having a lower % of the rAAV population.

The best parameter to track sample polydispersity, and what is an acceptable polydispersity level, depends ultimately on the sample type, the effect of the sample size and aggregates on the efficacy and safety of the formulation. Therefore, it needs to be derived on a case-by-case basis during drug development, while determining the critical to quality attributes. Batch-to-batch variability assessment and the acceptable batch values should then be controlled accordingly.

#### 3.1.5. What Can Be Learnt about the Polydispersity of the Main Population?

During development, it may be interesting to determine the polydispersity of a single particle population within a sample. Parameters such as either (i) percent polydispersity (%Pd) [62] which is calculated from (population peak width/population peak area) × 100 from DLS and MADLS or (ii) Mw/Mn [61] measured by SEC-SLS, can be used. In this context, for a population to be considered monodisperse, a common guideline is that the % polydispersity should be less than 20% [62], or for chromatography, the Mw/Mn is close to 1.00.

Among the MADLS measurements reported in Table 4, three samples present a monodisperse main peak (rAAV5 full, liposome and MVA). However, as already observed in Section 3.1.1, the MVA PSD determined by MADLS does not capture the full PSD if compared with NTA, only the populations at the top end. Therefore, the polydispersity of these peaks will not be discussed further here.

The liposome and the rAAV5 samples show <20%Pd for MADLS, but NIBS DLS data give a polydispersity slightly above, and indicate some polydispersity present in the sample, which is confirmed by the SEC-SLS data shown in Figure 3b. For the liposome data, MADLS can identify a small fraction of larger aggregates, which would contribute to a broadening of NIBS DLS data. The empty rAAV5 and the mRNA-LNP2 could be considered moderately polydisperse, since the value is close to 20%Pd for MADLS, but significantly higher when measured by NIBS DLS. Here, additional populations have been identified with higher resolution techniques, as confirmed by NTA data (Figure 2) for mRNA-LNP2 and by SEC-SLS (Figure 3) for the empty rAAV5. MADLS reports a higher peak polydispersity for mRNA-LNP 1. This is not surprising, as the MADLS data show a clear shoulder in the main population (Figure 2a).

SEC-SLS can resolve multiple populations and thus a polydispersity value can be calculated for each of the populations present in the sample. For both rAAV5 samples, after resolution of multiple populations with SEC, the Mw/Mn associated with the main population is very close to 1 (monodisperse).

In contrast, mRNA-LNP 1 and mRNA-LNP 2 show a Mw/Mn in the order of 1.1–1.15, indicating that they are composed of one single moderately polydisperse population of vesicles. This higher Mw/Mn can also be observed for the larger aggregates peak in rAAV5 empty sample. These in-depth findings about the different populations present in the sample would not have been possible without the increased resolution associated with the SEC separation prior to analysis of each population.

Significant information can be obtained by combining size and polydispersity metrics, whether it is identifying populations present in the sample, or understanding the composition of a single population, or using them as quality metrics during manufacture to ensure consistent production of these particles.

### 3.2. Particle Concentration of Viral Capside Titer

#### 3.2.1. What Concentration Range Can Be Measured for a Particular Vector?

The concentration of a sample can be assessed in different ways, ranging from mass-based measurements such as the concentration expressed in mg/mL measured by concentration detectors such as UV-Vis or refractive index measurements, to number-based particle concentration which reports how many particles are dispersed in the sample volume (particles/mL). In this section, the applicability of MADLS, NTA and SEC-SLS to measure particle concentration of some of the delivery vectors shown in the previous section is discussed.

**Figure 4:**
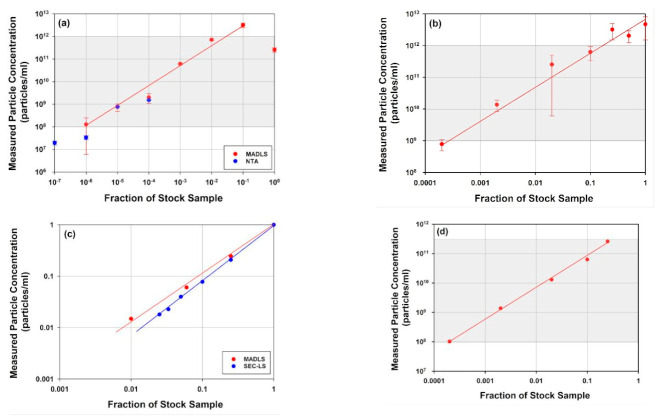
Examples of accessible concentration range for delivery vectors of different sizes. (**a**) Liposome sample (~100 nm) measured by MADLS and NTA; (**b**) LNP 1 (~87 nm) measured by MADLS; (**c**) full rAAV5 (~30 nm) measured by MADLS and SEC-SLS (the measurements were done on two different batches with different stock concentrations 8 × 10^13^ and 3.1 × 10^13^ particles/mL, respectively); (**d**) LNP2 (~104 nm) measured by MADLS. Indicated particle sizes (in brackets) are main population diameter in MADLS measurements. Shaded on each graph is the theoretical region for concentration based on the particle size and optical properties such as particle refractive index and absorbance.

In Figure 4, dilution series of full rAAV, two mRNA-LNPs of different sizes and a liposome sample measured with MADLS, NTA or SEC-SLS are shown. The accessible concentration ranges for MADLS for each of the samples, are highlighted as a shaded area in each figure. For the three larger samples, the liposome and the two mRNA-LNP samples, the accessible concentration range is 10^9^ to 10^12^ particles/mL, see Figure 4a,b,d. For the liposome sample and mRNA-LNP2, due to their larger size and therefore increased scattering, an order of magnitude lower is achievable, down into the order of 10^8^ particles/mL.

These two larger samples, which inherently scatter more (Figure 4a,d), both clearly show non-linear behavior at the highest sample concentration, resulting in an apparent decrease in the reported value at the maximum concentration. This is due to an apparent reduction in particle size as a consequence of multiple scattering effects being present at high concentrations (size data not shown) [38,59,60]. There is also an indication that this is observed for mRNA-LNP1 as the concentration flattens at the top end (Figure 4a).

The accessible concentration range for MADLS overlaps with the NTA concentration range of 10^7^ to 10^9^ particles/mL (Figure 4a) for the liposome sample, where NTA and MADLS concentration complement each other and partly overlap and extend the measurable concentration range.

In contrast with these larger samples, the much smaller rAAV sample requires higher concentrations for particle concentration measurements (Figure 4b), where both MADLS and SEC-SLS measurements are shown. Here, MADLS can measure over three orders of magnitude, 10^11^ to 10^14^ particles/mL, which overlaps neatly with the SEC-SLS determined particle concentration, which is in the range of 10^11^ to 10^13^ particles/mL.

These data demonstrate how light scattering-based techniques can provide orthogonal measurements of particle concentration for samples across a wide range of concentrations and particle sizes. The applicability of these measurements will depend on the stage of development as it often governs the amount of sample available for measurements. For synthetic vectors, that typically are >50 nm in diameter, the non-destructive MADLS measurements are often a quick screen for size and particle concentration, to understand formulation steps and their impact on the particle size distribution. In contrast, for viral vectors, where often the early stages of production mean very low concentration of vectors, NTA and its ability to access lower concentration ranges may be better suited for these samples. For the small rAAV samples, MADLS can be used as a rough screen for particle concentration, or total viral titer as it is often referred to, during purification stages, with its use being increased during latter stages of development.

#### 3.2.2. MADLS Extends the Measurement Range for Heterogenous Samples

**Figure 5:**
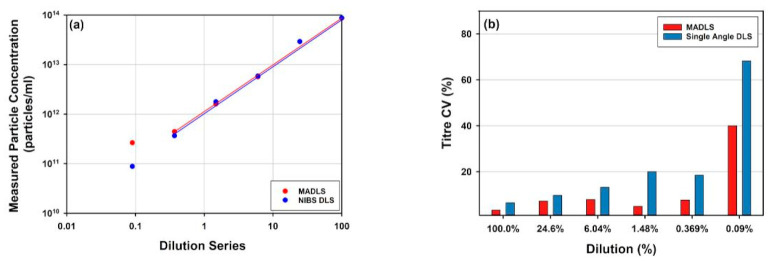

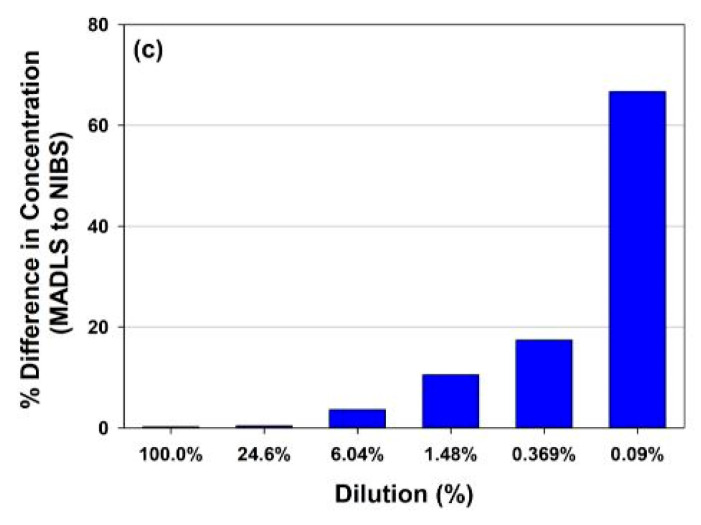
Comparison of rAAV5 concentrations measured using single-angle DLS (backscatter) or MADLS: (**a**) Total viral particle concentration for the sample across the dilution series; (**b**) Variability across repeat measurement for each dilution; (**c**) Deviation in concentration value determined with single-angle DLS versus MADLS measurements.

What are the benefits of MADLS vs. single angle DLS with similar functionality for particle concentration measurements? Figure 5 shows a comparison for the full rAAV5 sample, comparing MADLS particle concentration with particle concentration calculated in a similar manner from a single angle backscatter measurement. In Figure 5a, the dilution curves are shown for the same samples measured with the two methods, and they follow a similar pattern.

However, as seen in Figure 5c, the deviation between the two methods increases as the sample is diluted. It can also be seen that the variability in replicate measurements increases as the sample is diluted for single angle measurements, but not to the same degree for the MADLS particle concentration measurements (Figure 5b). As a sample is diluted, the relative signal arising from the sample decreases in comparison to the signal from the buffer, reducing signal to noise and making the measurements more sensitive to any noise (e.g., dust particles or small aggregate). A low number of larger particles also become visible in DLS/MADLS due to the dependency of the scattering to the sixth power of the diameter i.e., d^6^ [63]. At higher sample concentrations, the sample dominates the scattering derived from any dust present.

There is no real benefit in using MADLS compared to single angle measurements for monodisperse samples around the stock concentration (Figure 5). However, once the sample has slight polydispersity, the benefit of using MADLS becomes clear with the variability of the data being lower compared to single angle measurements. There is a limit to how polydisperse a sample can be before the MADLS concentration data start to vary significantly and this is critically dependent on the variability of the size measurement. This can be seen in the lowest concentrations of MADLS measurements (size data not shown) causing increased spread in concentration results. If the size peaks overlay for repeat measurements, then MADLS will work well, but if they start to vary, the variability will increase, with concentration variability often being a factor of 10 larger than size variability. This is the most critical factor in the repeatability of concentration data [38].

### 3.3. Identification of Components and Quantification of Payload

#### 3.3.1. Recombinant Adeno-Associated Viruses

**Figure 6:**
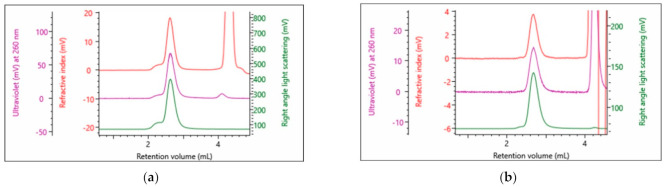
Multi-detection chromatogram of the different serotypes as separated on a Sepax SRT 500 and analyzed on an OMNISEC system: (**a**) rAAV9 (full); (**b**) rAAV2 (full).

The rAAVs discussed are utilized for cell gene therapy, whereby the particle is used to encapsulate the genetic material and acts as a vehicle to deliver the payload to the desired target. Some of the critical quality attributes (CQAs) such as % monomer and particle concentration have been introduced. In this study, SEC-SLS was shown to be able to determine both. Moreover, the use of inline RI, UV and SLS detectors in combination also allows for the quantification of the payload. In addition to the benefits illustrated in previous work [20], which outlines how this technique is generally less time-consuming, labor-intensive, more accurate and precise, the technique is also serotype independent. To further illustrate this, rAAV9 and rAAV2 were also studied. Figure 6 shows chromatograms of the full rAAV9 and rAAV2, respectively.

The compositional analysis discussed in the method section of this article was applied to the monomer peak in each of the empty and full samples of the rAAV5, rAAV9 and rAAV2 serotypes. This analysis was done on multiple injections to indicate the repeatability of the results (Table 7). The absolute molecular weights of each empty rAAV sample were 3.86, 3.80, and 3.62 MDa, respectively, meeting the expectations for the empty capsids of rAAV5, rAAV9 and rAAV2. The full samples had significantly higher molecular weights of 4.50, 4.53, and 4.51 MDa, respectively. These higher molecular weights indicate that the monomer now contains genetic material. The capsid % result indicates the percentage of protein by weight in the analyzed sample. The empty rAAV5 approaches 100% protein with the full rAAV5 being only 83.9 confirming it consists of 26.1% genetic material. Applying the compositional analysis on the samples also produces results for the samples’ CQAs. It shows the full rAAV5, rAAV9 and rAAV2 consist of 78, 77 and 80% filled monomer particles and have vp/vg ratios of 1.29, 1.30 and 1.25, respectively (Table 4). Additionally, the particle concentration is produced and is labelled total AAV titer.

Analyzing the monomer peak in each rAAV9 and rAAV2 nominally full and empty with the compositional analysis of the OMNISEC software, the percentage full, the vp/vg ratio and the particle titer are produced from a single measurement. The samples are simply differentiated by the software by applying the appropriate dn/dc and dA/dc values for the two components of each of the serotypes.

**Figure 7:**
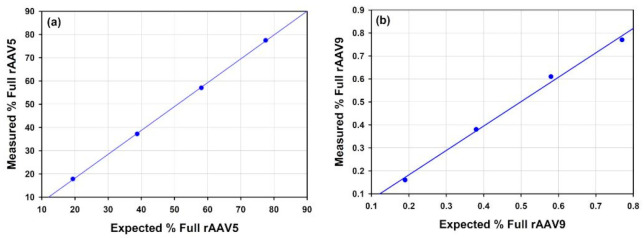
Charts show the measured percentage of full rAAV in comparison to the expected values based on mixing the nominally full and empty rAAV: (**a**) rAAV5, R^2^ = 0.000852; (**b**) rAAV9, R^2^ = 0.994974.

Empty and full rAAVs of a given serotype were then mixed to give 3 additional points that when measured would indicate how accurately the % full rAAV could be measured over a range of percentage full particles. The graphs presented below (Figure 7) illustrate that the measured full % follow the expected values produced from the mixing of the nominally full and empty samples.

**Figure 8:**
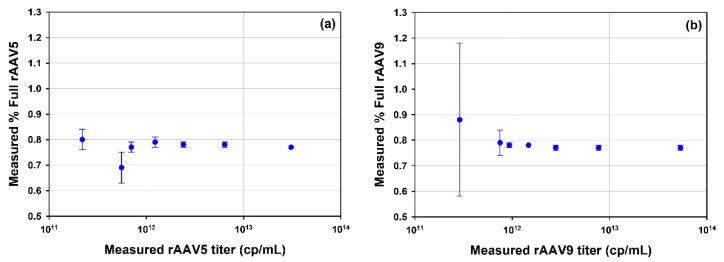
Plots of measured % full rAAV as a function of the measured rAAV titer (cp/mL); (**a**) rAAV5; (**b**) rAAV9.

A series of dilutions of the rAAV5 full and rAAV9 full samples were prepared to test the lower concentration limit for determining the % full rAAV (Figure 8). For both tested serotypes, the accuracy and repeatability of the measurement is well maintained until diluted to *circa* 1 × 10^12^ cp/mL. A greater degree of variation and reduced accuracy is observed at particle concentrations diluted below 1 × 10^12^ cp/mL.

#### 3.3.2. Lipid Nanoparticles

**Figure 9:**
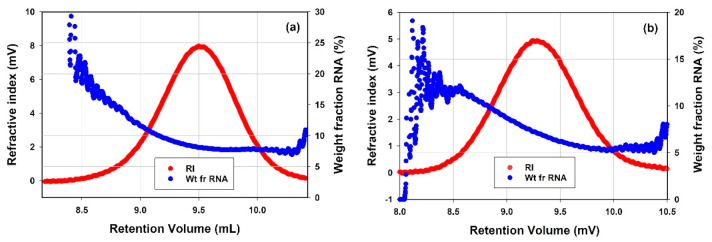
RI chromatograms of (**a**) mRNA-LNP1 and (**b**) mRNA-LNP2 overlayed with respective weight fraction of mRNA.

As shown earlier in this article, SEC of mRNA-LNPs separates the samples by size and the presence of different molecular weights was identified and quantified. Unlike the rAAV samples, the mRNA-LNP samples did not contain defined monomeric or oligomeric states, but rather each sample existed as a single mode containing a distribution of molecular weights. Additionally, the compositional analysis applied above to the rAAV samples can also be applied to the mRNA-LNPs by dividing them into two component parts: LNP and the genetic material. One major difference between the mRNA-LNP and rAAV samples is their size. Due to the larger size of the mRNA-LNPs, they scatter UV light in addition to absorbing UV light upon UV-based detection. The scattering is not insignificant and, by modelling the increased baseline levels caused by the particle scattering light at wavelengths where no absorption is expected (400−800 nm), the contribution of scattering at the wavelength at which compositional analyses were done can be accounted for. The primary results from the compositional analysis on the mRNA-LNP samples are the concentration of each component at each data slice of the distribution. They each may be summed to give the sub-total concentration of each component and a comparison of the two concentrations can give the weight fraction (%) of each component. mRNA-LNP1 was shown to have a weight fraction mRNA of 8.8 ± 0.25%, and mRNA-LNP2 7.6 ± 0.26%. Moreover, not only can the weight fraction (%) be produced from the sub-totals but also at each data slice. Presented below are the RI chromatograms of mRNA-LNP1 and mRNA-LNP2 (Figure 9a,b, respectively), and overlayed on each chromatogram is weight fraction of mRNA. This illustrates that there is not a defined amount of mRNA enclosed in each mRNA-LNP but rather a distribution of particles with differing quantities of mRNA for each of the mRNA-LNP samples tested.

### 3.4. Electrophoretic Light Scattering

In order to assess the robustness of the measurement protocol on different formulations, it is important to assess the variability from repeated measurements. In some cases, different behavior can be experienced even if measuring two formulations theoretically belonging to the same nanoparticle class. The results from ELS and DLS measurements of the two mRNA-LNP samples are summarized in Table 8. The results of both lipid nanoparticles show good repeatability for both average diameters and zeta potential means.

The zeta potential means for mRNA-LNP2 have smaller negative values (−6.63 mV) compared to mRNA-LNP1 (−19.5 mV). The sizes of both mRNA-LNP1 and mRNA-LNP2 samples also show excellent repeatability. The z-average diameters of the mRNA-LNP2 sample are larger compared to mRNA-LNP1 (102.1 nm compared to 69.7 nm). It is worth noting here that DLS measurements of free mRNA gave z-average diameters of around 50 nm (results not shown).

#### pH Titrations

**Figure 10:**
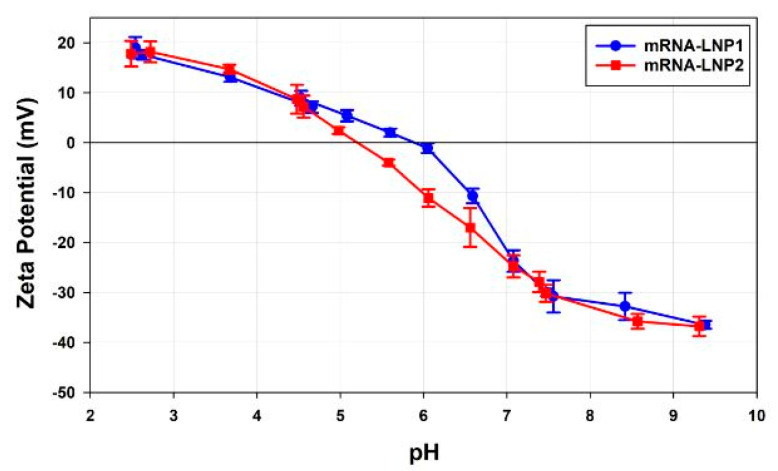
A plot of the zeta potential values (in mV) as a function of pH for mRNA-LNP1 and mRNA-LNP2 samples prepared in 10 mM NaCl.

Figure 10 shows plots of the zeta potential values as a function of pH for mRNA-LNP1 and mRNA-LNP2 samples prepared in 10 mM NaCl. Whereas the zeta potential values in acidic and alkali conditions are consistent, the isoelectric points (pI) of the two samples are different, with mRNA-LNP1 having a pI at pH 5.89 and mRNA-LNP2 at pH 5.21, respectively.

### 3.5. Thermal Stability by DLS Thermal Ramps and DSC

#### 3.5.1. Thermal Stability of rAAV5 by DLS Thermal Ramps and DSC

**Figure 11:**
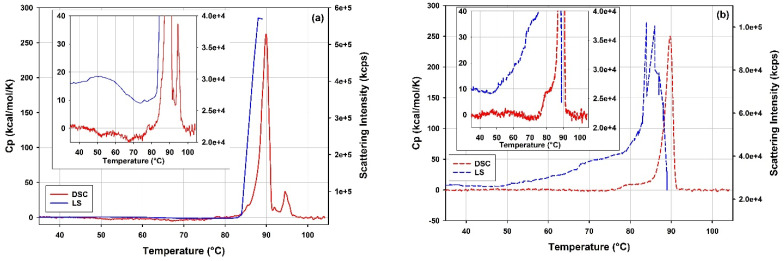
Overlay of the DSC and Light Scattering (LS) thermal ramp traces of (**a**) the full and (**b**) the empty rAAV5 samples. The inserts show the overlays in the temperature range preceding the main transition. The DSC data are corrected for the instrument blank and the baseline contribution and normalized by the VP protein content in the respective rAAV5 samples. The light scattering data from the thermal ramp DLS measurement is presented as derived count rate, DCR in kilocounts per second.

Figure 11 presents overlays of the DSC thermograms and the light scattering traces from DLS thermal ramps of the full and the empty rAAV5 samples across a broad range of temperatures. As previously described [20], the full and the empty rAAV5 show significantly different responses to thermal stress when studied with DLS and DSC.

Whilst the empty and the full rAAV5 samples show increased light scattering intensity around 45 °C, the empty rAAV5 sample appears to be more prone to thermal destabilization as, unlike the full rAAV5 sample, it displays a steady increase in the light scattering intensity. This accelerates in the temperature range corresponding to the pre-transition shoulder on the DSC thermogram of the empty rAAV5 sample in the range between 75 °C to 85 °C.

Apart from similar Tm values (cf. 89.43 and 89.68 °C) characteristic of the rAAV5 serotype [38,64] and the similar Δ*H*_tr_ values for the main transitions (cf. 2360 and 2260 kJ/(mole VP protein)), the empty and full rAAV5 samples show different responses to thermal stress according to the DSC data. A dip in the DSC signal can be observed on the thermogram of the full rAAV5 sample in the temperature range preceding the main peak. This deviating behavior is corroborated by the light scattering intensity data which also shows markedly different trends for the full and the empty rAAV5 samples in this temperature range (cf. inserts in Figure 11). In addition, a pre-transition shoulder at temperatures preceding the main transition is observed only on the DSC thermogram of the empty rAAV5 sample. The differences persist in the temperature range following the main peak, where an additional transition is detected for the full rAAV5 sample and centered around 94.7 °C. This additional higher temperature transition is markedly different from the Tm value specific to the rAAV5 serotype and could be attributed to structural transitions of ssDNA molecules released upon disintegration of the rAAV5 capsids [20].

**Figure 12:**
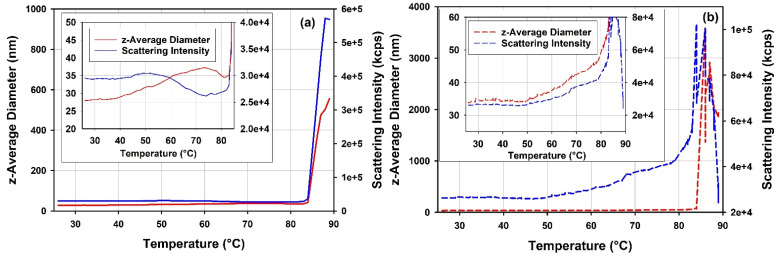
DLS thermal ramp data as overlays of the scattering intensities, DRC in kilocounts per second and z—average hydrodynamic diameter in nanometers as a function of temperature for (**a**) the full rAAV5 and (**b**) for the empty rAAV5.

Figure 12 shows overlays of light scattering intensities and z-average diameters of the full and the empty rAAV5 samples during DLS thermal ramps. The mean scattering intensity and z-average size of the empty rAAV5 samples increase in response to the temperature ramp. The temperature dependence of these parameters for the full rAAV5 appears to be complex, with a region where opposite trends are observed for the mean scattering intensity and z-average size (cf. inserts Figure 12). The difference in the response of the two rAAV5 samples to thermal stress becomes even more pronounced at temperature range >85 °C. This temperature range is accompanied by a fast increase of the mean scattering intensity and z-average size for the full rAAV5 sample, and a gradual increase of the mean scattering intensity and z-average size followed by a downturn for the empty rAAV5. The maximum value observed for the mean scattering intensity of the empty rAAV5 is significantly lower than the value observed for the full rAAV5. These trends are reproduced for diluted rAAV5 samples (data not shown). The overall changes in z-average size with temperature could contain a contribution from the changes in viscosity of the samples. However, as the same buffer was used for the two rAAV5 samples compared in these DLS thermal ramps, any changes in sample viscosity would originate from the changes in the state of the rAAV5 samples.

#### 3.5.2. Thermal Stability of mRNA-LNP1 and mRNA-LNP2 by DLS Thermal Ramps and DSC

The results of thermal stability of the mRNA-LNP samples are summarized in Table 9. Due to the complexity of the mRNA-LNP samples, the profiles of the thermograms are included as comparison of qualitative fingerprints specific to each sample, along with the corresponding numerical parameters such as integrated heat effect, temperature of thermal transition, T_m_ and temperature of onset of thermal transition, T_onset_. Repeat measurements were made with an interval of 9 days for the mRNA-LNP samples stored at 4 °C. Minor changes in the parameters of the thermal transitions can be observed between the repeat measurements. However, due to low sample concentration (0.994 mg/mL total lipid and 0.04 mg/mL mRNA for mRNA-LNP1 and 0.561 mg/mL total lipid and 0.023 mg/mL mRNA for mRNA-LNP2), the somewhat lower signal-to-noise ratio does not allow definite conclusions about significance of the differences observed for the repeated sample runs to be made.

**Figure 13:**
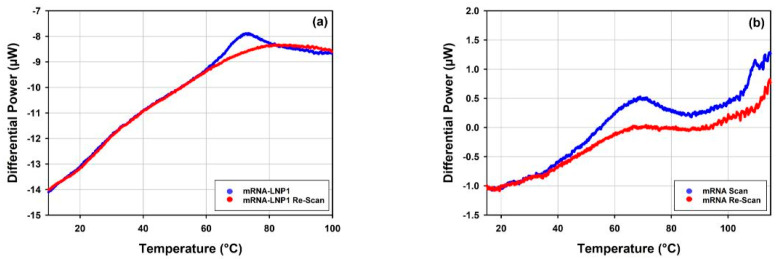
Overlays of the raw DSC data as traces of differential power, DP over temperature for a scan and a re-scan of (**a**) mRNA-LNP1 and (**b**) free mRNA samples.

All the mRNA-LNP samples were tested for reversibility of the thermal transitions by performing a re-scan following each DSC scan of the samples. Figure 13 shows overlays of the DSC data obtained for the scans and re-scans of mRNA-LNP1 and free mRNA samples. Prior to each scan and re-scan, samples were incubated for 5 min in the calorimetric measuring cell at the starting temperature of 10 °C.

One peak is well resolved on the raw DSC trace of the mRNA-LNP1 sample (Figure 13a), and the process associated with this peak appears to be irreversible on the time scale of the experiment (approximately 40 min from the onset of the peak and the end of the first scan) and not reproduced upon subsequent re-scan. This was the case for all the mRNA-LNP samples tested in this study (data not shown). On the contrary, the thermal transition associated with the peak at around 65 °C on the raw DSC trace of the free mRNA sample (Figure 13b), appears to show about 40% reversibility, whilst the thermal transitions observed above 100 °C is not reproduced upon the re-scan of the mRNA sample. The observed reversibility refers to the time span required for the cooling of the sample after the first scan and the start of the re-scan which is approximately 30 min for the DSC re-scans in this study.

**Figure 14:**
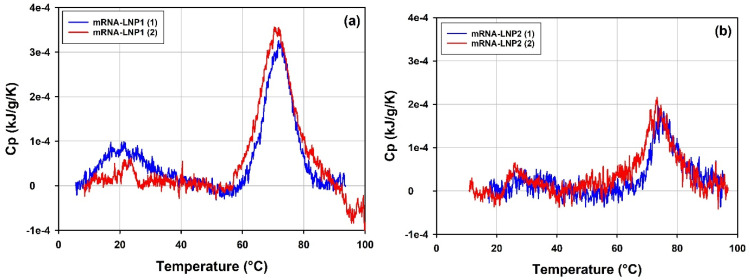
Overlays of the thermograms obtained for (**a**) mRNA-LNP1 and (**b**) mRNA-LNP2 batch 1 samples. DSC data corrected for the instrumental blank and the baseline and normalized for the total lipid concentration.

The results of the measurements on mRNA-LNP1 and mRNA-LNP2 samples are presented in Figure 14 and Table 8. mRNA-LNP1 (Figure 14a) and mRNA-LNP2 (Figure 14b) show well-reproduced DSC profiles with at least two transitions of significantly different peak amplitudes identified in a lower temperature range and in a higher temperature range. The amplitude of the transitions is consistently higher for the mRNA-LNP1 samples. This is possibly due to differences in the composition and concentration of the samples and/or different structural arrangements within mRNA-LNP particles.

**Figure 15:**
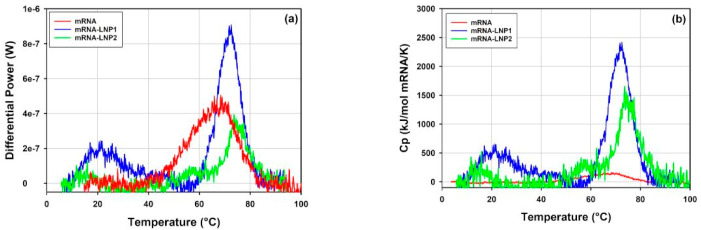
Overlays of the DSC traces of mRNA-LNP1, mRNA-LNP2 and free mRNA samples corrected for the instrumental blank and baseline and presented as (**a**) not normalized differential power and as (**b**) normalized per mole of mRNA for each sample and expressed as apparent excess heat capacity.

Figure 15 shows an overlay of DSC thermograms obtained for the free mRNA and the mRNA-LNP1 and mRNA-LNP2 samples in the temperature range between 10 and 100 °C. The first peak observed for the free mRNA (68 °C) overlaps with the main transitions observed on the DSC thermograms of the mRNA-LNP1 and mRNA-LNP2 samples (Figure 15a). The transitions correspond to heat effects of 0.371, 0.485 and 0.247 mJ for the free mRNA, mRNA-LNP1 and mRNA-LNP2, respectively. Notably, for the free mRNA sample, this heat effect corresponds to mRNA concentration of 0.32 mg/mL while the mRNA concentration in the LNP samples is about an order of magnitude lower (0.041 mg/mL for mRNA-LNP1 and 0.026 mg/mL for mRNA-LNP2). Therefore, the heat effects observed for the main transitions in mRNA-LNP1 and mRNA-LNP2 are significantly higher and cannot be accounted for by the heat of transition of the free mRNA only (Figure 15b). This large discrepancy could be caused by the presence of mRNA-cationic lipid complex and the likely linkage between the mRNA structural transitions and the disruption of interactions within the complex, with potential effects on the overall lipid assembly and phases.

**Figure 16:**
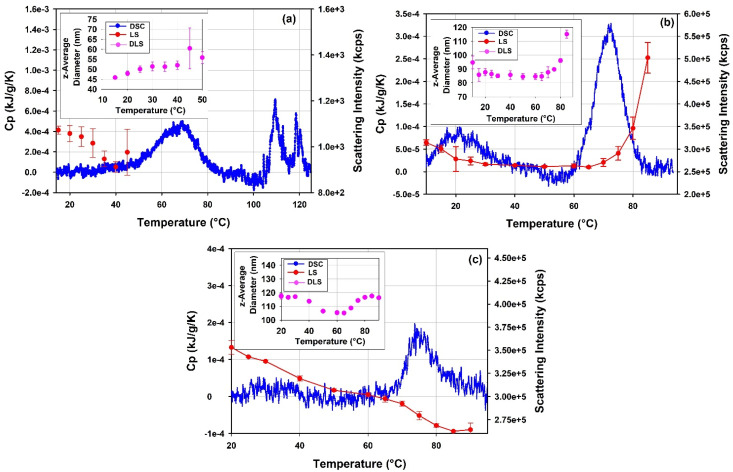
DSC thermograms overlaid on DLS thermal ramp data displayed as light scattering intensity vs. temperature for (**a**) the free mRNA, (**b**) mRNA-LNP1 and (**c**) mRNA-LNP2. The inserts present the dependence of the z-average diameter on temperature for the respective samples. To facilitate the comparison, DSC data are shown normalized per mass concentration of the sample, i.e., per gram of mRNA for the free mRNA sample and per gram of total lipids for mRNA-LNP1 and mRNA-LNP2, respectively.

The structural and compositional complexity of the mRNA-LNP particles is enhanced by the structural complexity of its components. mRNA molecules are known to be structurally flexible. Several thermal transitions can be resolved on the DSC trace of the free mRNA sample (Figure 16a). They could be attributed to rearrangements of the tertiary and the secondary structure.

To further explore the details of the thermal transitions and the stability of the free mRNA and mRNA-LNP samples observed in DSC, thermal ramp DLS measurements have been conducted on these samples. The results are presented in Figure 16. Trends in the light scattering intensity data obtained for the mRNA-LNP1 and the free mRNA report changes in the sample properties and are in good agreement with the ranges of thermal transitions mapped by DSC.

DLS thermal ramp data for the free mRNA point to a moderate size increase from 46 to 56 nm in the temperature range from 10 °C to 50 °C. This change in size could be related to a structural rearrangement in the mRNA sample. Further, the opposite trends observed (cf. insert Figure 16a) between the light scattering intensity and z-average size for the free mRNA sample at temperatures <50 °C suggest changes in the packing density and refractive index of the sample. The data from DLS thermal ramp of the mRNA-LNP samples (Figure 16b,c) show good agreement between the trends in light scattering intensity and z-average size of the sample up to ca 65 °C. Notably, the two observables show steady decrease in the temperature range between ~10 °C and ~35 °C for mRNA-LNP1 and between ~15 °C and ~50 °C for mRNA-LNP2, and then remain invariant with temperature until about 60 °C. This temperature dependence maps well on the temperature range of thermal transitions detected by DSC for the mRNA-LNP1 and mRNA-LNP2 samples. It follows that the lower temperature thermal transition observed for these samples in DSC is accompanied by a decrease in the z-average size of the mRNA-LNP particles up to about 35 °C and 50 °C for mRNA-LNP1 and mRNA-LNP2, respectively, whilst the higher temperature transition is accompanied by an increase in size for both mRNA-LNP samples. The steep increase in z-average diameter for the mRNA-LNP1 sample in the range corresponding to the higher temperature DSC transition is accompanied by marked increase in the light scattering intensity which can be expected from first-principle relation of these parameters. On the contrary, the pronounced increase in size of mRNA-LNP2 sample in the range corresponding to the higher temperature DSC transition is accompanies with decrease in the light scattering intensity in the absence of a significant sample loss from the solution. (Figure 16c). The nature of these temperature-induced transitions is not possible to discern from DSC and DLS alone. However, the agreement between the temperature ranges detected by light scattering and calorimetric techniques (Figure 16) provides a strong argument in favor of the presence of a structural change and its dependence on the type of the cationic lipid used in LNP formulation.

## 4. Discussion

The performance and applicability of the six light scattering and calorimetric techniques to measure the physical−chemical properties of multiple samples of relevance to viral and nucleic acid-based vaccines, including rAAVs and mRNA-LNPs, have been tested in this study.

The results covered in the previous section and Table 10 have underlined some method specific considerations about the applicability and limitations of these methods, as well as highlighted benefits from their orthogonal use which is discussed in more detail in this section.

### 4.1. Size Distribution and Sample Polydispersity

The fate of delivery vectors inside the body is dependent on several factors, one of them being its size [65]. The size is not only critical for the end function, but the PSD also reports on any potential instability in the sample, where degradation such as fragmentation or aggregation has occurred due to any external stresses, such as storage conditions or processing steps. As demonstrated in Section 3.1 and summarized in Table 10, there are multiple analytical methods available to characterize the PSDs of different delivery vectors.

The choice of the analytical technique used will depend on both sample specifics such as the measurement purpose, the stage of development and sample volume available, but also on the technique’s availability and applicability (measurement size and concentration range). In the case of larger delivery vectors, such as lipid nanoparticles (LNPs and liposomes), as well as larger viral particles, such as the Modified Vaccinia Ankara (MVA) virus, which together covers a size range from 50 to 400 nm, NTA, MADLS and DLS are well capable of measuring their size and size distribution [57,58]. Depending on the polydispersity of the sample, as shown in Section 3.1.2, it may be necessary to apply a separation technique such as FFF or SEC before the measurement for a more detailed understanding of the size distribution.

For the analysis of batch-to-batch consistency, the cumulants analysis is commonly used to track any changes in the sample, as the z-average size and the PdI are the most robust parameters generated with DLS [30,58]. DLS is better suited than NTA to cover a wide range of sizes, from smaller particles to larger aggregates for monodisperse samples and samples with a relatively low polydispersity where a second population of larger material may be present. Combination of information from multiple angles, as is done with MADLS, gives consistent detection of low numbers of aggregates in these formulations. This is because the larger particles scatter more light in the forward direction and are not as easily detected with non-invasive back scatter (NIBS), as shown in the liposome sample in Figure 2e or mRNA-LNP1 in Figure 2a.

Small lipidic or protein-based particles <50 nm cannot be detected with NTA [57]. The technique is also less sensitive in detecting small populations of larger aggregates, because of it being a number-based technique. However, NTA provides higher resolving power [57] than both DLS and MADLS and can therefore provide information on sample polydispersity and resolving multiple populations in the main particle size distribution peak (see mRNA-LNPs and MVA vector examples in Figure 2). MADLS provides improved resolution compared to DLS, but not as high as NTA, as seen in the mRNA-LNP1 data where NTA can identify secondary populations present in the sample. In the MVA sample, NTA is also capable of showing the increased resolution in the size distribution [57].

For a deeper insight into the size distribution, higher resolution methods using a separation method, such as SEC or FFF, can improve the understanding of the full population present in the sample, as demonstrated in the two batches of rAAV5 samples (Figure 3 and Table 5 and Table 6). Here, it is clear how MADLS is a very sensitive screening tool for the presence of small amounts of larger material. However, it cannot resolve close populations such as monomers and small oligomers, but it requires a separation technique such as SEC-SLS to detect and quantify small oligomers.

The heterogeneity or polydispersity of drug delivery vectors can also be important in their development as it provides information on sample stability and process reproducibility. An acceptable polydispersity will depend ultimately on the sample, with some samples being very monodisperse, whereas others are inherently polydisperse. The important factor is the control and maintenance of polydispersity to be similar across batches or tests. The key is to decide what is important for the sample and its intended function.

### 4.2. Particle Concentration

Particle concentration measurements using light scattering techniques provide orthogonal measurements to mass-based measurements and allow for quantification of vector concentrations. This is particularly useful when they are multi-component systems and mass-based measurements may be representative of the bulk of the material present, and not the vectors themselves. Similar limitations exist for size measurements.

The accessible concentration range varies with delivery vector size and the requirements of the analytical technique. NTA has an optimal concentration range ~10^7^ to 10^9^ particles/mL and can be used for delivery vectors with a size from about 50 nm to around 500 nm in diameter. In contrast, the accessible concentration range for MADLS is limited by the amount of light scattered by the sample and is therefore directly related to the size and the refractive index of the delivery vector [38]. Importantly, for robust measurements, it should be verified that the particle system is stable upon any required dilution, often done simply by measuring the particle size distribution repeatedly over time and monitoring the sample for any trends.

SEC-SLS can measure concentrations of particles that are capable of being separated on a SEC column, so typically smaller than 200 nm in diameter. The OMNISEC SEC system uses information from multiple detectors to produce various results with the particle concentration results determined from data obtained with the light scattering detector and differential refractometer. The limitation of the results is therefore dependent on the two detectors. The signal observed from the RI detector is dependent on the concentration of the sample, and the light scattering detector signal is dependent on the molecular weight and concentration. For vector type samples, their relatively high molecular weight leads to intense light scattering signals, which leads to the RI detector becoming the limiting detector in particle concentration calculations.

### 4.3. SEC-SLS

For gene therapy-based vaccine products to be marketed, the characterization of the vector must meet certain specifications that are related to vector identity, purity, and potency is a prerequisite [14]. The quantification of the payload present in both rAAV and LNP based delivery vectors is key to dosing and potency analysis of the vaccine vectors [15]. Without implementing an accurate fit for purpose analytical technique, clearly the dose that patients receive is impossible to control and therefore the results from potency assays can be variable and difficult to interpret. This makes it obligatory to determine the quantity of the payload contained within the vaccine vectors. SEC-SLS, as noted above, has been shown to be an appropriate technique to separate, characterize and quantify the populations within a single sample determination, whereby characterization and quantification reveal the molecular weight, the molecular weight distribution and the %weight fraction each population accounts for in the sample. Moreover, the implementation of compositional analysis has also emerged to further bolster the value of this technique by quantifying the payload in the delivery vectors with the knowledge of simple parameters of a given sample’s parameters.

#### 4.3.1. AAV

Compositional analysis was used to characterize %full in rAAV samples of different serotypes. The full rAAV5, rAAV9 and rAAV2 samples were analyzed and the %full was determined to be 77.49, 76.97 and 80.04, respectively, with a high degree of accuracy as illustrated by % RSDs of less than 1.2% (Table 6). It was demonstrated that the capabilities of SEC-SLS to the analysis of these rAAV samples, consistently determining genome titer and therefore %full rAAV and cp/vg was possible at different levels of concentrations as the fullrAAV5 and rAAV9 samples were analyzed for a series of dilutions from greater than 1 × 10^13^ cp/mL to less than 1 × 10^12^ cp/mL. To confirm the reliability of the technique to determine the %full, rAAV were analyzed across more than one serotype, full rAAV and empty rAAV of serotypes 5 and 9 were mixed in three ratios in order to create three additional samples of each serotype with known or expected %full AAV. The calculated %full rAAV results were plotted against the expected values in Figure 7a,b. The results show good alignment between the calculated and the expected values for both serotypes. This proof of concept further confirms the credibility of the use of SEC-SLS for this sample type.

#### 4.3.2. LNPs

The compositional analysis was also applied to the characterization of mRNA-LNPs as the quantification of the payload encapsulated in the particle is one of the CQAs. Similarly to the AAV samples, it is an attribute used for determination of dosing and in potency analysis. The mRNA payload of the mRNA-LNPs is presented in the results differently compared to how the load of ssDNA cargo is presented for AAV samples. Rather than doing further calculations, the weight fraction of mRNA can be used as is to keep track of the overall amount of mRNA being delivered in a given dose. The two mRNA-LNP samples were characterized with similar values of the mRNA payload, where mRNA-LNP1 was shown to have a weight fraction mRNA of 8.8 ± 0.25%, and mRNA-LNP2 was shown to have a decreased weight fraction of mRNA of 7.6 ± 0.26%. In addition to the average results the weight fraction of mRNA was presented as a function of the retention volume (Figure 9). In Figure 9a,b, the weight fraction mRNA is seen to change with retention volume. It is observed that in both mRNA-LNP samples the larger, higher molecular weight particles which elute at earlier retention volumes contain more mRNA. The mRNA payload decreases as the particles continue to elute. This provides an insight into the range of weight fraction of mRNA present in the mRNA-LNP samples.

### 4.4. Zeta Potential Discussion

The size, polydispersity and zeta potential of lipid nanoparticles can be used as critical quality attributes of products. Stability can be studied by monitoring changes in particle size and polydispersity. For example, measurements taken over time will indicate the presence of any aggregation which might be present and help predict sample shelf life. In addition, changes to the chemistry of the dispersant (for example pH) can be performed to elucidate how sensitive the particles are to the solution they are dispersed in.

Knowledge of the size and zeta potential of lipid nanoparticles can help to predict their fate in vivo [66,67]. The effect of different formulations of a lipid nanoparticle can be determined by measuring their size and zeta potential, as can any subsequent modification of the lipid nanoparticle surface.

Table 7 summarizes the particle sizes and zeta potential values of mRNA-LNP1 and mRNA-LNP2 prepared in PBS buffer. The particle sizes and zeta potential values of the mRNA-LNP1 and mRNA-LNP2 samples both show good repeatability. Other studies have shown that different formulations have shown different transfection efficacy (paper in preparation). Therefore, the repeatability of the particle size and zeta potential results of lipid nanoparticles are key in understanding formulation stability.

The lipid nanoparticles used in this study have four main components, a neutral phospholipid, cholesterol, a PEGylated lipid and an ionizable cationic lipid. The role of the PEGylated lipid is to control particle size and prevent aggregation during storage through a steric stabilization mechanism. The ionizable lipid is positively charged containing ionizable amine groups. The use of this ionizable lipid is two-fold. Firstly, it interacts with the mRNA during nanoparticle formation, and secondly, it aids membrane interaction [68,69]. A recent study has shown that the ionizable lipid is mainly located inside the core of the lipid nanoparticles and the interaction with mRNA can influence the ionizability of the lipid [18].

The stability of the lipid nanoparticles could be lost in different ways, including aggregation, fusion, leakage of encapsulated mRNA or changes in the concentration of components at the surface of the nanoparticles. The consistent z-average diameters and zeta potential means obtained from repeat measurements of mRNA-LNP1 and mRNA-LNP2 summarized in Table 7 confirm that neither aggregation nor fusion are present and that the lipid nanoparticles are stable during the measurement period. Any change in zeta potential values across repeat measurements would suggest that changes in the composition of surface components may be occurring. Repeat measurements are important to perform to ensure that there are no stability issues present

Figure 10 summarizes the zeta potential values (in mV) as a function of pH for mRNA-LNP1 and mRNA-LNP2 prepared in 10 mM NaCl. The zeta potential values of both mRNA-LNP1 and mRNA-LNP2 show similar values at both low and high pH values. However, they exhibit different isoelectric points (pI) with mRNA-LNP1 having a pI at pH 5.90, whilst mRNA-LNP2 has a pI at pH 5.21. This difference in the pI values is due to the different ionizable lipids which are present in both lipid nanoparticle samples. The pI values of the two mRNA-LNP samples do not correspond with the pKa values of the different ionizable lipids present in the nanoparticles but does correlate with the lipid to mRNA ratio and are consistent with other observations reported [25].

Performing pH titrations is important in understanding how sensitive samples are to changes in pH conditions and how this could influence formulation stability.

### 4.5. Thermal Stability by DLS Thermal Ramps and DSC

Complex structures of viral- and lipid-based vectors are formed through, and maintained by, interactions between their multiple components. Various stress conditions can impact physical and chemical integrity of the constituents of these biomolecular assemblies and affect the network of their interactions, and therefore the structure, stability and ultimately the function of the entire delivery vector.

Change in temperature is a common stress factor for biologics. Measurements of the impact of thermal stress on viral and lipid-based vectors help to understand the behavior and structure of these biomolecular assemblies in solution and inform on rational approaches to their development and design of stable liquid formulation.

DSC thermograms provide means to explore thermal unfolding or phase transitions of biomolecules such as proteins, DNA, RNA and lipids and their assemblies. DSC traces, along with the corresponding values of total heat effect, enthalpy of transitions, Δ*H*_tr_, the midpoint temperature of a thermal transition, T_m_ and the temperature of the onset of thermal transition, T_onset_, serve as sample-specific fingerprints enabling detailed characterization and comparison of thermal stability and Higher Oder Structure of biomolecules in solutions.

DLS thermal ramp data report changes in the mean light scattering intensity of a samples as a function of temperature. It also provides information on the size and the polydispersity of a sample in solution at each temperature point in a sequence of DLS measurements collected during the thermal ramp. A change in the light scattering intensity of a sample can arise from a change in sample concentration, sample size and homogeneity as well as a shift in the refractive index of the sample and/or dispersant caused by change of temperature.

An increase in the light scattering intensity could often be attributed to aggregation, whilst a decrease in the light scattering intensity could be associated with loss of sample from solution due to sample precipitation and surface adsorption, or a decrease in the refractive index of the sample due to change of structure, packing and sample density.

Greater insights and convergence of evidence can be expected when thermal stability profiles of rAAV and mRNA-LNP samples are mapped orthogonally through DLS thermal ramps and DSC.

DSC thermograms provide a way of mapping thermal transitions in these complex samples and help to evaluate structural stability and phase behavior of their constituents.

In this study, a combination of DSC and DLS thermal ramps was used to explore thermal stability and temperature-induced transitions of rAAV5 and mRNA-LNP samples.

#### 4.5.1. Thermal Stability of rAAV5 

Stability information of AAV vectors is required to drive their design and formulation development. Viral capsids need to be stable enough to protect their genome load and delivery to the target. At the same time, sufficient conformational lability is required to enable the release of the cargo at a replication site, where structural change appears to be required for genome release. The mechanism of AAV vector uncoating and genome release, and conditions affecting it in vivo and upon handling and storage, remain a subject of investigation. Based on the published DSF and DSC [29,64] data on thermal stability of AAVs AAV undergoes a thermal transition with a characteristic T_m_ value. The transition is related to the capsid disassembly process and its T_m_ serves as an indicator of AAV serotype. T_m_ values are generally quite similar for empty and full AAV capsids of one serotype, and they have no clear correlation with capsid dynamics, capsid uncoating, and genome release. New biophysical approaches are required to inform on the stability of AAV vectors to support their design and formulation development.

The thermal stability of empty and full rAAV5 samples evaluated by DSC, and the parameters of the thermal transitions detected for the two samples, have been previously reported [20].

In this study, the details of the observed thermal transitions by combining the DSC results with the trends in particle size and light scattering intensity obtained from DLS thermal ramps conducted on the empty and the full rAAV5 samples have been explored. The combination of these orthogonal techniques, and the published data on the thermal stability of AAV vectors collected with atomic force microscopy, transmission electron microscopy, biochemical assay and fluorimetric detection [70,71], enabled more detailed mapping of structural changes occurring in the rAAV5 samples during temperature stress.

The differences in response of the full and the empty rAAV5 samples to thermal stress are evident from the results of the two orthogonal techniques (Figure 11 and Figure 12). These marked differences point to the significance of the product purity, such as relative content of the full capsids to the overall stability profile of rAAV5 batches. The onset of the thermally induced aggregation for both the full and the empty rAAV5 samples is observed at temperature well below the T_m_ characteristic of unfolding of rAAV5 serotype (~90 °C). Whilst the aggregation progresses in the empty rAAV5 sample at temperatures > 45 °C, for the full rAAV5 it overlaps with another process on-going in the temperature range between 50 °C and 75 °C and is accompanied by the release of heat (Figure 11a) and the opposite trends in the size and the light scattering intensity (Figure 12a). The latter, in the absence of the sample loss from solution, can be attributed to structural changes leading to reduction of the refractive index of the rAAV5 sample, such as ejection of ssDNA from the capsids in a fraction of the sample. Additional measurements with dedicated techniques are required to explore this process in detail and to confirm and quantify the extent of ssDNA ejection in the full rAAV5 sample at temperatures preceding capsid disintegration. The finding points to the potential of DLS thermal ramps and DSC for label-free characterization of AAV stability in solution in response to thermal stress and formulation or storage conditions.

#### 4.5.2. Thermal Stability of mRNA-LNP1 and mRNA-LNP2 

Lipid nanoparticles present a promising alternative to viral delivery vectors for vaccines and therapeutic drugs with benefits of cell-free production process and capability for rapid upscale. However, a better understanding of their complex structure and behavior in solution is required to control production and to secure designed function and stability on storage.

DSC is a well-established technique to monitor phase transitions of lipids [72], structural transitions of nucleic acids, their complexes with cationic lipids [48,73,74,75,76] and thermal stability of lipid-based delivery vectors, [12,55].

In this study, DSC has been used to explore thermally induced structural transitions of the free mRNA and the mRNA-lipid complexes in mRNA-LNP1 and mRNA-LNP2 samples. Sample complexity makes detailed analysis and data interpretation challenging, and a combination of DSC with DLS size and thermal ramp measurements to increase the level of insight and reliability of the data interpretation have been used.

Of the components making up, mRNA-LNP1 and mRNA-LNP2, at least two, DSPC and mRNA, can undergo thermal transitions when studied free in solutions [46,72]. Ali et al. [72] reported two thermotropic phase transitions for DSPC liposomes based on DSC measurements—a pretransition at 48.1 °C and the main phase transition at 53.4 °C accompanied by ΔH = 8.9 kJ/mol and ΔH = 48.1 kJ/mol, respectively. No prior data existed on structural transitions and DSC analysis of the mRNA used for preparations of mRNA-LNP samples in this study.

RNA structural transitions can be triggered by a diversity of external factors such as temperature, pH, salt concentration and binding of ligands [46]. RNA molecules are known to be structurally flexible and can be organized in diversity of secondary and tertiary structures [46,47,48]. Large RNA molecules may be built of several domains which can undergo cooperative and non-cooperative transitions. Thermal stability of RNA and DNA can be analyzed with UV, circular dichroism spectroscopy and nuclear magnetic resonance as well as by following activity for catalytically active RNA molecules. DSC provides means of exploring these transitions in detail and provides resolution comparable to that of UV-based assays but not limited by high absorbance limitations and hypo- and hyperchromicity issues. Thermal transition profiles of RNA samples from DSC can contain multiple peaks and be quite complex [75,77]. DSC thermogram of the FLuc mRNA (Figure 15a) used in this study is rather complex with at least three transitions (T_m1_ = 67.2 °C, T_m2_ = 109.5 °C and T_m3_ = 118.4 °C, respectively).

Comparison of the DSC results obtained for the free mRNA and mRNA-LNP samples (Figure 14) suggests that the presence of multiple components in the LNP formulations, their relative distribution and interactions affect the behavior of the individual components. The main transition detected for the free mRNA sample is not reproduced on the DSC thermograms of the mRNA-LNP samples, which show main peaks at T_m_ values shifted to higher temperatures. Additionally, the heat effects observed for the main transitions in mRNA-LNP1 and mRNA-LNP2 are comparable in size to the heat effect associated with transition in the free mRNA sample. However, if these heat effects are compared on a per mole mRNA basis, the normalized heat effects corresponding to mRNA-LNP transitions are significantly higher than the heat of transition of the free mRNA (Figure 15). This is likely due to the dynamics of mRNA-cationic lipid complexes and lipid phases within the sample such as hexagonal and lamellar phases described by Kotlover et al. [75], Middaugh and Ramsey [74] and Larson et al. [19]. The results of this limited study suggest that the contributions of the mRNA and lipid transitions to the DSC thermogram profile of the mRNA-LNP preparations are different from an additive combination of transitions of individual components.

Reversibility is another important aspect of the structural transitions observed in DSC. Irreversibility of the thermal transitions of mRNA-LNPs observed in this study (Figure 12a) is likely due to the complexity of structure and interaction networks within the mRNA-lipid complexes and lipid phases resulting in kinetically controlled states and slow (if any) relaxation to the original structures [19]. This might be the case for the mRNA which demonstrated approximately 40% reversibility of the 1st thermal transition and no reversibility for the higher temperature transition (Figure 12b) on the time scale of 30 min. In a structure as complex as mRNA-LNP, reversibility of the structural transitions is not implied and, if present, can be expected to be kinetically controlled and may depend on the direction of the change.

## 5. Conclusions

Characterization of nucleic acid-based vaccines delivered as viral and non-viral vectors requires fit-for-purpose analytics and a significant level of method understanding for robust results and adequate interpretation of experimental findings collected for these highly complex samples.

Here, we demonstrate how first-principle, label-free biophysical techniques can be applied to characterization of physical and chemical quality attributes of viruses and lipid nanoparticles without the need for dedicated reagents and calibration reference standards. These results have shown that there is often a need for complementary and orthogonal techniques to obtain a fuller picture of a delivery vector, and the techniques to be used may vary with the nature of the vector, especially based on its size or components.

## Figures and Tables

**Table 1:** Technologies used in characterization of viral and lipid-based vectors *.

Property	Relevant Technology	References
Capsid/particle size	DLS, SEC/AF4-SLS, NTA, cryo-TEM	[7,12,19,20]
Capsid titer or particle count	MADLS, SEC/AF4-SLS, NTA	[7,12,20]
Fragmentation	SEC/AF4-SLS	
Aggregate formation	DLS, MADLS, NTA, TEM, AF4-SLS	[7,12,20]
Composition:		
Percentage of genome-containing virus particles/%full analysis	SEC/AF4-SLS, Anion exchange chromatography, analytical ultracentrifugation, native MS, ELISA, qPCR	[7,21]
Encapsulation level	LC-UV-Vis, fluorescence assays, gel electrophoresis	[22]
Lipid quantification	LC coupled with Charged aerosol detector or Evaporative light scattering detector or MS	[7,12,22,23,24]
Charge	ELS	[7,25]
Binding interaction	Isothermal titration calorimetry	[26,27]
Thermal stability	DSC, DLS thermal ramp, DSF	[20,28,29]

* Table contains more techniques than used in this study.

**Table 2:** Summary of the NTA detection settings for mRNA-LNP samples.

Sample	Camera Level	Detection Threshold	Software Version
Values used for LNP1 and LNP2	16	5	NTA software version 3.4
Liposomes	13–16 *	4–6 *	NTA software version 3.2
Modified Vaccinia Ankara (MVA)	14	5	NTA software version 3.4

* Setting varies with sample concentration.

**Table 3:** Viral and non-viral delivery vectors used in this study.

Delivery Vector	Formulation Description	Payload/Transgene ^1^	Expected Size Range, Diameter
Full rAAV5	Recombinant Adeno-associated virus serotype 5 with transgene	pFB-GFP ssDNA 2544 bases	25–35 nm
Empty rAAV5	Recombinant Adeno-associated virus serotype 5 without transgene	-	25–35 nm
mRNA-LNP1	LNP 1 (MC3) 994.4 μg/mL (Total lipid mass)	0.041 mg/mL Fluc mRNA 1929 bases	50–120 nm
mRNA-LNP2	LNP2 (SM102) 560.7 μg/mL (Total lipid mass)	0.026 mg/mL FLuc mRNA 1929 bases	50–100 nm
Liposome	HSPC/CHOL liposomes	-	60–110 nm
MVA	Modified Vaccinia Ankara virus (attenuated)	linear dsDNA *ca* 180 kbp ^2^	100–400 nm (elongated)

^1^ Nomenclature differs between non-viral and viral application area, respectively. ^2^ kilo base pairs.

**Table 4:** Summary of size results for viral and non-viral vectors. Numbers between brackets are relative standard deviation RSD). NTA is not applicable (n/a) for rAAV5 samples due to their small size. When no second peak is identified “-” is inserted.

Sample: (Repeat Measurements per Aliquot)	Z-Average (Cumulants Analysis, nm)	Peak 1 mean (NNLS analysis, nm)	Peak 2 Mean (NNLS Analysis, nm)	Peak 1 Mean (MADLS Analysis, nm)	Peak 2 Mean (MADLS Analysis, nm)	Size Distribution Mode (NTA, nm)	Size Distribution Mean (NTA, nm)
LNP 1 (5)	87.1 ± 5.5 (6.3%)	81.7 ± 2.9 (3.5%)	1930 ± 1922 (99.5%)	75.0 ± 6.8 (9.1%)	246 ± 179 (73%)	64 ± 6 (10%)	95 ± 2 (2.1%)
LNP 2 (5)	104.3 ± 2.3 (2.2%)	116.4 ± 8.7 (7.5%)	5021 ± 5 (0.01%)	105.9 ± 6.3 (5.9%)	420 ± 88.0 (21%)	82 ± 25 (31%)	116 ± 15 (13%)
Liposomes (5)	100.1 ± 3.6 (3.6%)	105.5 ± 4.0 (3.8%)	-	98.8 ± 3.5 (3.5%)	452 ± 6 (1.3%)	85.5 ± 3.6 (4%)	89.4 ± 0.4 (0.4%)
Modified Vaccinia Ankara (MVA) (5)	250 ± 3.0 (1.2%)	323 ± 15.0 (4.6%)	4877 ± 42 (0.9%)	178 ± 11 (6.2%)	428 ± 11 (2.6%)	119 ± 14 (11%)	186 ± 7 (3.6%)
rAAV5 full 1 (5)	25.4 ± 0.1 (0.3%)	26.7 ± 0.2 (0.9%)	-	25.4 ± 0.1 (0.3%)	-	n/a	n/a
rAAV5 empty 1 (5)	29.5 ± 0.2 (0.5%)	33.2 ± 0.4 (1.3%)	-	30.7 ± 0.4 (1.3%)	-	n/a	n/a

DLS measurements on rAAV samples are shown in Table 6.

**Table 5:** Population data for rAAV5 empty and full by SEC-SLS. Batch 1 ^1^ and Batch 2 ^2^ were analyzed using different columns.

	Monomer	Mw (g/mol)	Mw/Mn	Frac. of Sample (%)	Peak Conc. (mg/mL)
rAAV5 Empty ^1^	Monomer	3.79 × 10^6^ ± 2.89 × 10^4^	1.004 ± 0.0020	91.23 ± 1.09	0.341 ± 0.0142
	Dimer	7.1 × 10^6^ ± 5.58 × 10^5^	1.011 ± 0.0049	6.55 ± 0.72	0.025 ± 0.0018
	Aggregates	2.2 × 10^7^ ± 6.65 × 10^6^	1.19 ± 0.0934	2.22 ± 0.49	0.008 ± 0.0017
rAAV5 Full 1	Monomer	4.52 × 10^6^ ± 7.34 × 10^4^	1.001 ± 0.0007	94.02 ± 1.26	0.411 ± 0.0059
	Dimer	5 × 10^6 3^ ± 1.34 × 10^6^	1.10 ± 0.1133	2.48 ± 0.69	0.011 ± 0.0030
	Aggregates	7.8 × 10^6 3^ ± 2.63 × 10^5^	1.12 ± 0.0442	3.49 ± 0.57	0.015 ± 0.0025
rAAV5 Empty ^2^	Monomer	3.63 × 10^6^ ± 2.27 × 10^4^	1.012 ± 0.0026	98.01 ± 0.32	0.256 ± 0.0045
	Dimer	7.1 × 10^6^ ± 5.39 × 10^5^	1.006 ± 0.0049	1.99 ± 0.32	0.005 ± 0.0009
rAAV5 Full 2	Monomer	4.43 × 10^6^ ± 2.09 × 10^4^	1.007 ± 0.0019	96.53 ± 0.30	0.191 ±0.0115
	Dimer	7.5 × 10^6^ ± 1.76 × 10^5^	1.04 ± 0.0119	3.15 ± 0.17	0.006 ± 0.0001
	Aggregates	2.7 × 10^9 3^ ± 4.67 × 10^9^	1.32 ± 0.4908	0.33 ± 0.25	0.01 ± 0.0005

^1^ SEC was performed with a Superose 6 25/300 on batch 1. ^2^ SEC was done on a Sepax SRT 500 4.6 × 300 mm on batch 2. ^3^ Approaching limit of quantification leads to results susceptible to baseline instability and or reduced signal to noise ratio.

**Table 6:** Polydispersity of delivery vectors determined by DLS, MADLS, SEC-SLS and NTA. Some of the parameters are specific for the analytical technique, with some techniques using the same way of calculating sample polydispersity. n/a indicates where data is not available. * For the MVA sample, where it was determined that the MADLS data only captured the top end of the sample distribution, the MADLS polydispersity data have been excluded. For the other techniques, the main peak is considered to be the peak around 200 nm in diameter. The span is calculated from the 90th, 50th and 10th percentiles of the size distribution, using either NIBS (volume transformation), MADLS or NTA.

Delivery Vector (Number of Repeat Measurements)	Polydispersity Index (PdI) ^1a^ (%Pd)	Span (D90–D10)/D50)	Main Population Peak Polydispersity (%Pd)	Mw/Mn ^4^	% Monomer ^4^
rAAV5 full (6)	0.03 ± 0.01 ^1a^ (17)	0.70 ± 0.01 ^1c^, 0.42 ± 0.02 ^2c^ n/a ^3^	24.0 ± 0.6 ^1b^, 15.0 ± 0.8 ^2^	1.004	94.0
rAAV5 empty (6)	0.12 ± 0.02 ^1a^ (34)	0.90 ± 0.02 ^1c^, 0.61 ± 0.05 ^2c^ n/a ^3^	35.5 ± 1.7 ^1b^, 23.2 ± 2.4 ^2^	1.001	91.2
LNP 1 (5)	0.325±0.004 ^1a^ (57)	6.95 ± 4.24 ^1c^, 0.92 ± 0.17 ^2c^ 0.97 ± 0.19 ^3^	43.9 ± 5.3 ^1b^, 33.0 ± 9.4 ^2^	(1.13)	(100)
LNP2 (5)	0.159±0.017 ^1a^ (40)	1.29 ± 0.17 ^1c^, 0.70 ± 0.16 ^2c^ 0.98 ± 0.32 ^3^	39.7 ± 3.4 ^1b^, 24.0 ± 3.9 ^2^	(1.16)	(100)
Liposome (5)	0.032 ± 0.016 ^1a^ (18)	0.75 ± 0.05 ^1c^, 0.46 ± 0.04 ^2c^ 0.38 ± 0.02 ^3^	24.1 ± 1.7 ^1b^, 16.0 ± 1.9 ^2^	n/a	n/a
MVA (10/5 **)	0.227 ± 0.020 ^1a^ (48)	1.85± 0.14 ^1c^, Excluded *^2c^ 1.04 ± 0.07 ^3^	53.6 ± 4.0 ^1b^, excluded *^2^	n/a	n/a

^1a^ DLS Cumulants analysis, ^1b^ DLS non-negative least squares (NNLS) analysis, ^1c^ DLS Volume transformation NNLS analysis, ^2^ MADLS analysis intensity, ^2c^ MADLS analysis volume-transformation, ^3^ NTA, ^4^ SEC-SLS, ** for calculation of span.

**Table 7:** Quantitative parameters for monomers in each the Empty rAAV5, Full rAAV5, Empty rAAV9, Full rAAV9, Empty rAAV2 and Full rAAV2 samples and the percentage relative standard deviations of the results.

	Mw (g/mol)	Mw/Mn	Wt Fr (Capsid) (%)	% Full AAV	vp/vg Ratio	AAV Titer (vp/mL)
Empty rAAV5	3,860,000	1.011	99.82			3.71 × 10^13^
% RSD	2.1	0.34	0.01			4.0
Full rAAV5	4,502,000	1.008	83.9	77.49	1.291	3.08 × 10^13^
% RSD	0.47	0.21	0.13	0.47	0.47	5.7
Empty rAAV9	3,798,000	1.007	99.83			4.34 × 10^13^
% RSD	0.79	0.18	0.12			5.1
Full rAAV9	4,526,000	1.015	84.13	76.97	1.299	5.35 × 10^13^
% RSD	0.43	0.19	0.19	1.2	1.2	2.8
Empty rAAV2	3,619,000	1.002	99.85			4.10 × 10^12^
% RSD	7.3	0.22	0.18			11
rAAV2 Full	4,513,000	1.005	83.28	80.04	1.249	1.05 × 10^13^
% RSD	0.90	0.07	0.13	0.62	0.62	3.8

**Table 8:** Zeta potential mean values (in mV) and z-average diameters (in nm) for the mRNA-LNP1 and mRNA-LNP2 samples measured in PBS using the diffusion barrier method.

Measurement	Zeta Potential (mV)		z-Average Diameters (nm)	
	mRNA-LNP1	mRNA-LNP2	mRNA-LNP1	mRNA-LNP2
1	−20.0	−5.35	68.9	99.9
2	−18.0	−6.30	69.7	102.6
3	−20.5	−8.23	70.0	102.8
4	-	-	69.9	102.3
5	-	-	70.2	103.1
Mean	−19.5	−6.63	69.7	102.1
Standard Deviation	1.32	1.47	0.50	1.29

**Table 9:** Summary of the results of DSC measurements of mRNA-LNP samples. Repeat measurements were made with an interval of 9 days.

Sample	Run #	T_m1_, C	T_m2_, C	Total Area, mJ
mRNA-LNP1	1	20.9	71.7	0.633
	2	21.4	71.1	0.687
mRNA-LNP2	1	25.6	75.0	0.204
	2	24.6	73.6	0.197

**Table 10:** Sample attributes measured in this study, with corresponding techniques and measurands reporting on the attributes.

Attribute	Measurement Techniques	Measurand—Parameter Abbreviation (unit)	Sample Information Required?	Sample Applicability
PSD	DLS	Z-average diameter—Dh (nm)	Dispersant viscosity and refractive index	From 0.3 nm to 10–20 µm ^1^.
	NTA	Number based size distribution mean or mode (nm)	Dispersant viscosity	From 10 nm to 2 µm ^1^.
	MADLS	Hydrodynamic diameter—Dh (nm)	Dispersant viscosity and refractive index, Particle absorbance and refractive index	From 0.3 nm to 500 nm ^1^.
	SEC-SLS/UV or SLS/RI	Molecular weight—(g/mol)	Particle dn/dc and dA/dc	<200 nm
Polydispersity	DLS (Cumulants Analysis)	Polydispersity Index—PdI	Dispersant viscosity and refractive index	Same as size measurements
	(DLS NNLS analysis)	Peak polydispersity—%Pd	Dispersant viscosity and refractive index (for volume and number transformations: Particle absorbance and refractive index)	
	NTA	Span	Dispersant viscosity	
	MADLS	Span	Particle absorbance and refractive index	
	SEC-UV or SEC-RI	Mw/Mn		
Particle concentration/Viral capsid titer	MADLS	Particle concentration (particles per mL)	Dispersant viscosity and refractive index, Particle absorbance and refractive index	
	NTA	Particle concentration (particles per mL)		
	SEC-SLS/UV or SLS/RI	Particle concentration (particles per mL)	Particle dn/dc or dA/dc	
Surface charge	ELS	Zeta potential (mV)	Dispersant viscosity	
Thermal stability	DSC	Tm, Tonset, ΔH, thermogram profile		
	DLS	Trend in light scattering and size	Dispersant viscosity ^2^	
Drug payload	SEC-SLS/RI/UV		Vehicle and drug’s dn/dc and dA/dc	

^1^ Depends on particle material, ^2^ Often corrected for temperature change by instrument software.

## Data Availability

The data presented in this study are available on request from the corresponding author. The data are not publicly available due to privacy reason.

## Abbreviations

AF4—asymmetric-flow field-flow fractionation; AFM—atomic force microscopy; CQAs—critical quality attributes; CHOL—cholesterol; DLS—dynamic light scattering; DRC—derived count rate; DSC—differential scanning calorimetry; DSF—differential scanning fluorimetry; DSPC—1,2-distearoyl-sn-glycero-3-phosphocholine; ELS—electrophoretic light scattering; HSPC—hydrogenated soybean phosphatidylcholine; LC-MS—liquid chromatography with mass spectrometry; LC-UV/Vis—liquid chromatography coupled to ultraviolet–visible detection; RNA-LNP—lipid-based nanoparticles for nucleic acid delivery; MADLS—multiangle dynamic light scattering; MALS—multiangle light scattering; MC3—dilinoleylmethyl-4-dimethylaminobutyrate; MVA—Modified Vaccinia Ankara; NIBS—non-invasive backscatter; NNLS—non-negative least squares; NTA—nanoparticle tracking analysis; PBS—phosphate buffered saline; PdI—polydispersity index; PFU—plaque forming units; PSD—particle size distribution; RI—refractive index; rAAV—Recombinant adeno-associated virus; SANS—small angle neutron scattering; SAXS—small angle x-ray scattering; SEC-SLS—size exclusion chromatography coupled to static light scattering detector; SLS—static light scatteing; SEC-MALS—size exclusion chromatography coupled to multiangle scattering detector; SEM—scanning electron microscopy; TEM—transmission electron microscopy; UV–Vis—ultraviolet–visible detection.
